# Ageing and rejuvenation models reveal changes in key microbial communities associated with healthy ageing

**DOI:** 10.1186/s40168-021-01189-5

**Published:** 2021-12-15

**Authors:** Jongoh Shin, Jung-Ran Noh, Donghui Choe, Namil Lee, Yoseb Song, Suhyung Cho, Eun-Jung Kang, Min-Jeong Go, Seok Kyun Ha, Dong-Ho Chang, Jae-Hoon Kim, Yong-Hoon Kim, Kyoung-Shim Kim, Haiyoung Jung, Myung Hee Kim, Bong-Hyun Sung, Seung-Goo Lee, Dae-Hee Lee, Byoung-Chan Kim, Chul-Ho Lee, Byung-Kwan Cho

**Affiliations:** 1 Department of Biological Sciences and KI for the BioCentury, Korea Advanced Institute of Science and Technology, Daejeon, 34141 Republic of Korea; 2 Laboratory Animal Resource Center, Korea Research Institute of Bioscience and Biotechnology, Daejeon, 34141 Republic of Korea; 3 Metabolic Regulation Research Center, Korea Research Institute of Bioscience and Biotechnology, Daejeon, 34141 Republic of Korea; 4 Immunotherapy Research Center, Korea Research Institute of Bioscience and Biotechnology, Daejeon, 34141 Republic of Korea; 5 Synthetic Biology & Bioengineering Research Center, Korea Research Institute of Bioscience and Biotechnology, Daejeon, 34141 Korea; 6 Healthbiome Co., Ltd., Daejeon, 34141 Republic of Korea

**Keywords:** Ageing, Rejuvenation, *Akkermansia muciniphila*, Shotgun metagenomics, 16s rRNA sequencing

## Abstract

**Background:**

The gut microbiota is associated with diverse age-related disorders. Several rejuvenation methods, such as probiotic administration and faecal microbiota transplantation, have been applied to alter the gut microbiome and promote healthy ageing. Nevertheless, prolongation of the health span of aged mice by remodelling the gut microbiome remains challenging.

**Results:**

Here, we report the changes in gut microbial communities and their functions in mouse models during ageing and three rejuvenation procedures including co-housing, serum-injection and parabiosis. Our results showed that the compositional structure and gene abundance of the intestinal microbiota changed dynamically during the ageing process. Through the three rejuvenation procedures, we observed that the microbial community and intestinal immunity of aged mice were comparable to those of young mice. The results of metagenomic data analysis underscore the importance of the high abundance of *Akkermansia* and the butyrate biosynthesis pathway in the rejuvenated mouse group. Furthermore, oral administration of *Akkermansia* sufficiently ameliorated the senescence-related phenotype in the intestinal systems in aged mice and extended the health span, as evidenced by the frailty index and restoration of muscle atrophy.

**Conclusions:**

In conclusion, the changes in key microbial communities and their functions during ageing and three rejuvenation procedures, and the increase in the healthy lifespan of aged mice by oral administration of *Akkermansia*. Our results provide a rationale for developing therapeutic strategies to achieve healthy active ageing.

Video abstract

**Supplementary Information:**

The online version contains supplementary material available at 10.1186/s40168-021-01189-5.

## Introduction

Health span is determined by the interactions between genetic and environmental factors [1, 2]. In particular, age-related low-grade chronic inflammation, called inflammageing [3], contributes to several age-related disorders such as cell senescence, autophagy dysfunction, chronic activation of the inflammasome, DNA damage accumulation and gut microbiota dysbiosis [4]. Moreover, as ageing progresses, the increase in intestinal permeability due to deterioration of the intestinal barrier function enhances the changes in the composition of intestinal bacteria and the production of bacteria-derived substances and causes systemic inflammation in a self-sustaining loop [5].

Both humans and laboratory animal models considerably alter the composition of intestinal bacteria as ageing progresses, and these changes affect the health and longevity of the host [6]. In line with this, faecal microbiota transplantation (FMT) of the gut microbiome from aged mice into germ-free, young mice further exacerbates inflammation [7]. Previous studies have repeatedly shown that the longevity of centenarians is positively related to the abundance of beneficial commensals [8]. For instance, *Akkermansia muciniphila* (AK) induces mucus production in the gut, which is critical for supporting intestinal integrity and other beneficial symbioses [9, 10]. The outer membrane protein AMUC_1100 of AK particularly improves gut barrier function and reduces endotoxemia in obese mice by stimulating TLR2 signalling [11]. These studies have suggested that beneficial commensals, such as *Akkermansia*, *Bifidobacterium* and *Christensenellaceae*, can be used as probiotics to improve various age-related symptoms [12]. Interestingly, heterochronic parabiosis allows young and aged mice to be surgically combined to achieve remarkable regenerative effects in aged tissues [13, 14]. Although the details are unidentified, it is predicted that soluble circulating factors and cells are delivered from young mice via blood circulation due to fatigue, and enhance immune function and inhibit inflammation [15, 16]. Nevertheless, the ageing process and changes in the gut microbiome with various rejuvenation methods have not yet been compared, and the prolongation of the health span of aged mice using beneficial gut microbes has not yet been described.

Here, we performed a systemic investigation of the changes in key gut microbial communities and their functions in mouse models during ageing and rejuvenation procedures, including co-housing, serum-injection and parabiosis. Moreover, we further evaluated the rejuvenation effects of the gut microbiota on the ageing-associated phenotype during the three rejuvenation experiments. Our results showed that the age-related microbiome and gastrointestinal disorders are reversible. Metagenomic data underscore the importance of the high abundance of *Akkermansia* and butyrate biosynthesis pathways in young and rejuvenated mouse groups. Furthermore, with an understanding of the relationship between ageing and the gut microbiome, the oral administration of *Akkermansia* ameliorates senescence-related phenotypes in intestinal integrity, muscle function and immune system in aged mice and thereby extending health span.

## Methods

### Animals

C57BL/6J mice were purchased from The Jackson Laboratory (Bar Harbor, ME, USA) and were maintained at the Korea Research Institute of Bioscience and Biotechnology (Daejeon, Korea). Mice were housed in a constant 12-h light/dark cycle with food and water ad libitum. All animal experiments were approved by the Institutional Animal Care and Use Committee (IACUC) of the KRIBB (KRIBB-AEC-18211) and were performed in accordance with the Guide for the Care and Use of Laboratory Animals published by the US National Institutes of Health.

### Co-housing and serum injection models

Young (4 months old, *n* = 9) and aged (18 months old, *n* = 9) mice were used for the co-housing experiment. Three young mice were co-caged with three aged mice for 6 weeks (three independent experiments). For the serum injection experiments, pooled mouse serum was collected from young (4–5 months old) mice. Serum was collected after blood was allowed to clot on ice for 30 min. The clots were removed by centrifugation. All serum aliquots were stored at − 80 °C until use. Young [5 months old; iv 8 (Y→Y), *n* = 10; iv 16 (Y→Y), *n* = 5] or aged [20 months old; iv 8 (Y→A), *n* = 10; iv 16 (Y→A), *n* = 5] mice were systemically treated with serum (100 μL per mouse) isolated from young mice by intravenous injection into the tail vein eight times (for 3 weeks) or sixteen times (for 6 weeks).

### Parabiosis model

Parabiosis surgery was performed as described previously [17]. Under ketamine-xylazine anesthesia and after clipping hair and prepping with betadine, a lateral longitudinal incision was made along the opposing sides of the two mouse groups. Two animals were connected through the elbow and knee joints, as well as the skin. This joining prevents extension of the skin and, therefore, causes less pain and complications. Young mice (5–6 months old) and aged mice (22–23 months old) were used in this study. Isochronic pairings (young-young or aged-aged) and heterochronic pairings (young-aged) of parabiosis were established. The parabiotic pairs were divided into three groups: (i) iso-young (young mice from isochronic young pairs, *n* = 6); (ii) hetero-chronic including hetero-young (young mice from heterochronic pairs, *n* = 5) and hetero-aged (aged mice from heterochronic pairs, *n* = 4); and (iii) iso-aged (aged mice from isochronically aged pairs, *n* = 8). After the suturing procedure, the mice were monitored daily for 6 weeks for signs of pain and distress. Body weight was recorded once per week. Circulatory exchange between parabiotic pairs was confirmed by injecting Evans blue dye [18]. Four hundred microliters of 0.5% Evans blue dye, in Hanks’ salt solution, was injected into one counterpart of parabiotic pairs through the tail vein. Blood was collected by retro-orbital bleeding at different time points (0, 0.5, 1, 24 and 48 h) after injection. The shared blood circulation of parabionts was determined by separating serum from the blood by centrifugation, diluting the sera (1:50), and measuring the absorbance at 620 nm.

### 16S ribosomal RNA and metagenomic sequencing

Snap-frozen mouse colon contents were stored at − 80 °C. Metagenomic DNA was extracted using the DNeasy PowerSoil DNA Pro Kit (QIAGEN, Hilden, Germany), as previously described [19]. The quality and concentration of the extracted DNA were measured using gel electrophoresis and the Qubit dsDNA HS Assay Kit (Thermo Scientific, Rockford, IL, USA) on a Qubit 3.0 fluorometer, respectively. All 16S ribosomal RNA (rRNA) gene sequencing libraries were prepared according to the 16S Metagenomics Sequencing Library Preparation protocol (Illumina, San Diego, CA, USA). The V3–V4 region of the 16S rRNA gene was amplified using region-specific primers that were compatible with the Illumina Nextera XT index and sequencing adapters. The PCR products were purified using AMPure XP beads (Beckman Coulter, CA, USA). The quality and quantity of 16S rRNA libraries were assessed using gel electrophoresis and a Qubit dsDNA HS Assay Kit on a Qubit 3.0 fluorometer, respectively. All sequencing libraries were quantified using the KAPA Library Quantification Kit for Illumina Platforms (Roche, Basel, Switzerland). Sequencing was performed on a MiSeq and HiSeq 2500 sequencing platform (Illumina, San Diego, CA, USA) using a 2 × 250 cycle protocol. For metagenomic sequencing, all libraries were prepared using the Nextera XT DNA Library Preparation Kit (Illumina, San Diego, CA, USA). For metagenome library construction, metagenomic DNA was subjected to 12 PCR cycles, according to the manufacturer’s recommendations. The amplified libraries were purified using AMPure XP beads. The quality and quantity of metagenomic libraries were assessed using an Agilent 2200 TapeStation (Agilent Technologies, Santa Clara, CA, USA) and the Qubit dsDNA HS Assay Kit on a Qubit 3.0 fluorometer, respectively. All sequencing libraries were quantified using the KAPA Library Quantification Kit for Illumina platforms. Sequencing was performed on a HiSeq 2500 sequencing platform using a 2 × 250 cycle protocol.

### Microbial community profiling

The QIIME2 pipeline (version 2018.2.0) was used to process raw paired-end reads according to a previously suggested workflow [20]. Reads were trimmed, dereplicated, combined and chimeras were removed using DADA2 [21] (qiime dada2 denoise-paired). The resulting reads were grouped into operational taxonomic units (OTUs) based on 99% sequence similarity (amplicon sequence variants). For ageing samples in mice of different ages (0, 4, 20, 50 and 100 weeks old), 14,527,091 pair-end reads of each 250-nucleotide sequence were generated, resulting in 5172 OTUs after quality control. For samples in three rejuvenation mouse models (co-housing, parabiosis, and serum injection), 18,897,496 pair-end reads of each 250-nucleotide sequence were obtained, resulting in 13,732 OTUs after quality control. For samples of oral administration of *Akkermansia*, 11 control aged mice and 11 *Akkermansia*-administered aged mice were used, obtaining 2,824,613 pair-end reads of each 250-nucleotide sequence, resulting in 3494 OTUs after quality control. In three separate analyses, gut microbiome samples were rarefied to 9644 reads (ageing analysis), 2783 reads (rejuvenation analysis) and 7125 reads (analysis of *Akkermansia* administration), the minimum number of reads per sample for each analysis. For phylogenetic diversity analyses, alignment of OTU sequence (qiime alignment ‘mafft’ and ‘mask’) and construction of phylogenetic tree (qiime phylogeny fasttree) were performed. Alpha-diversity analyses were performed based on several metrics (Shannon’s diversity index, observed OTUs and Faith’s PD) through core-metric analyses of QIIME2. Beta-diversity metrics were calculated based on the Jaccard, Bray-Curtis, unweighted UniFrac and weighted UniFrac distances. Additional 2D principal coordinate analysis (PCoA) plots for beta diversity metrics were generated using QIIME1 (make_2d_plots.py). Taxonomic classification was analysed using Greengenes 13_8 (Greengene), which contains the V3–V4 regions, using a pre-trained naïve Bayes classifier (qiime feature-classifier). To analyze taxonomic differences in bacterial abundance, linear discriminant analysis (LDA) effect size (LEfSe) was used [22], which uses the factorial Kruskal-Wallis sum-rank test (α = 0.05) to identify discriminative bacterial taxa.

### Metabolic function profiling

Before downstream metagenomic data analysis, raw reads with low-quality bases and adaptor sequences were filtered and trimmed using the CLC Genomics Workbench 6.5.1 (QIAGEN). The following parameters were applied for trimming: limit = 0.05, maximum two ambiguous nucleotides allowed. The resulting FASTQ sequence files were uploaded to the Metagenomics Rapid Annotation using Subsystems Technology (MG-RAST) server version 4.0.3 [23]. Uploaded reads were denoised and normalised, and sequencing artifacts and host (*Mus musculus*) DNA were removed using the MG-RAST pipeline as previously described [24]. Data representing each taxon and metabolic functions were obtained by annotating the query reads against the Kyoto Encyclopedia of Genes and Genomes (KEGG) Orthology [25], RefSeq and SEED subsystem database tools [26], with a minimum identity cutoff of 80%, a maximum *e* value cutoff of 10^−5^ and a minimum alignment length cutoff of 15 base pairs for RNA and 15 amino acids for proteins (default parameter). For statistical analysis of differential taxonomic abundance and metabolic functional potential, normalised count data were processed using DESeq2 [27]. Pairwise LEfSe was used to identify differentially abundant KEGG modules in the microbiome samples [22]. The KEGG modules with LEfSe values greater than two at a *P* value < 0.05 were considered statistically significant.

### Treatment with Akkermansia muciniphila

To investigate whether AK prolongs the lifespan, aged mice (24–25 months old, *n* = 20/group) were treated daily with *Akkermansia muciniphila* (AK, 4.9 × 10^8^ CFU per 150 μL per day) until they all died. For the anti-ageing experiment, aged mice (20–21 months old) were used in this study. The vehicle group (*n* = 10) was fed a culture medium (BTTM), and the AK-treated group (*n* = 11) was treated with AK grown on BTTM. Mice were treated daily with an oral administration of AK at the same concentration as that in the lifespan experiment, and treatment was continued for 36 weeks. Body weights were measured weekly.

### Quantification of frailty with the clinical FI

Frailty in aged mice was quantified using a slightly modified FI [28]. Twenty-seven health-related variables that provided information about activity levels, hemodynamic status, body composition and metabolism were used. Clinical assessment included evaluation of the integument, musculoskeletal system, vestibulocochlear/auditory systems, ocular and nasal systems, digestive system, urogenital system, respiratory system, signs of discomfort, body weight and body surface temperature. All clinical frailty assessments were performed by an experienced investigator. Table S7 lists the clinical signs of the deterioration/deficits evaluated in this study. To establish baseline clinical assessment techniques, young adult mice with few signs of clinical deterioration were initially observed and evaluated. The severity of each deficit was rated on a simple scale. A score of 0 was assigned if there was no sign of a deficit, a score of 0.5 indicated a mild deficit and a score of 1 was assigned for a severe deficit.

### Novel object recognition tests

The novel object recognition test was performed as previously described [29]. Mice were individually habituated to a testing chamber (40 × 20 × 20 cm^3^) with no objects for 5 min and then placed in a testing chamber for 10 min with two identical objects (familiar, acquisition session). The mice were then returned to their cages. One day later, the mice were placed back into the testing chamber in the presence of one of the original objects and one novel object (novel, recognition session) for 10 min. The original objects were cylindrical wooden blocks with a diameter of 10 cm (height) × 2 cm. The novel object was a 10 × 2.5 × 2 cm rectangular wooden block. The acquisition and recognition sessions were video-recorded, and an observer who was blinded to the drug treatment scored the time spent exploring the objects. The chambers and objects were cleaned with ethanol between trials. Exploration was defined as sniffing and touching the object with the nose and/or forepaws. Sitting on the object was not considered an exploratory behavior. A discrimination index (DI) was calculated for each animal and expressed using the following formula: [time (number) of contacts with the novel object–time (number) of contacts with the familiar object]/[time (number) of contacts with the novel object + time (number) of contacts with the familiar object) on day two.

### Grip strength and muscle weight measurements

The grip strength test was conducted with a grip strength machine (CCE, Bioseb, Vitrolles, France; 10 × 16 cm test grid). Mice were allowed to hold on to a metal grid with four paws and were gently pulled backward by the tail until the animals could no longer hold the grid. Each mouse was given five trials, and the average values were used to represent the muscle grip strength of an individual mouse. The investigator was blinded to the animal treatment.

### Blood and tissue sampling

Six weeks after the establishment of each experimental model, blood samples were collected from the orbital venous sinuses of each mouse. Plasma was prepared by centrifuging the blood at 10,000×*g* for 5 min at 4 °C and stored at − 80 °C until subsequent assays. After exsanguination, the mice were sacrificed by cervical dislocation. Intestine and muscle samples were collected for histological analysis, immediately immersed in liquid nitrogen, and stored at − 80 °C for further analysis.

### Plasma lipopolysaccharide analysis

Plasma LPS concentration was measured using a quantitative sandwich enzyme-linked immunosorbent assay kit (Cusabio, Houston, TX, USA). Briefly, a monoclonal antibody specific for LPS was pre-coated onto a microplate. Standards and plasma samples were applied to the wells so that any LPS present would bind to the immobilized antibody. After washing away any unbound substances, an enzyme-linked polyclonal antibody specific for LPS was added to the wells. Following this, a substrate solution was added for colour development (blue), in proportion to the amount of LPS bound in the initial step. The colour development was stopped with the stop solution, and the intensity of the colour (yellow) was read at 450 nm. The values are expressed in ng mL^−1^.

### Plasma corticosterone analysis

Corticosterone concentrations were measured using an ELISA kit purchased from Abcam (catalog number: ab108821). A 100-fold diluted plasma sample from each experiment was used, and assays were performed according to the manufacturer’s recommendations. Briefly, 25 μL of standards or samples were added to each well of the microtiter plate. Immediately, 25 μL of biotinylated corticosterone was added to each well, and the microtiter plate was shaken for 2 h at room temperature. After the plate was washed with the wash solution, 100 μL of the streptavidin–peroxidase conjugate was added to each well, and the plate was incubated for 30 min at room temperature. After the final wash step, 50 μL of chromogen substrate was added until the optimal blue colour density developed. The optical density (O.D.) of corticosterone was measured at 450 nm using a plate reader immediately after the reaction was terminated by adding 50 μL of the stop solution. The corticosterone concentration was calculated according to the standard curves.

### Quantitative reverse transcription PCR

Total RNA was prepared from intestinal samples using TRIzol reagent (Invitrogen). Quantification and integrity analysis of total RNA was performed using a NanoDrop spectrophotometer. cDNA was synthesised by reverse transcription, and quantitative reverse transcription PCR (qRT-PCR) was performed as previously described [30]. 18S rRNA was used as the housekeeping gene. The sequences of the primers used for qRT-PCR are listed in Table S8.

### Histology

At necropsy, intestinal samples were immediately fixed in 10% formaldehyde, embedded in paraffin and cut into 4 μm slices. The slides were stained with hematoxylin and eosin (H&E). Then, histological sections were viewed at 400× magnification, and images were obtained using a microscope (Olympus BX51, Tokyo, Japan). For goblet cell staining, deparaffinized and rehydrated sections were stained using PAS reaction. The number of goblet cells was expressed as the total number of PAS-positive cells per well-preserved crypt in the colon.

### Immunohistochemistry

Paraffin sections of the colon samples were incubated with a monoclonal rat anti-Ki 67 antibody (DAKO), biotin-conjugated polyclonal rabbit anti-rat IgG (Vector Laboratories, Burlingame, CA, USA) and streptavidin horseradish peroxidase conjugates at room temperature for 1 h. Signals were detected using the Vectastain ABC reagent (Vector Laboratories), and the slides were washed with phosphate-buffered saline (PBS). The 3,3′-diaminobenzidine chromogen was used for signal development. The sections were counterstained with hematoxylin. The average number of Ki-67-positive cells expressed on crypt base columnar (CBC) cells was determined by counting well-preserved crypts in the colon.

### Immunofluorescence staining of muscle tissue and size quantification

Mouse tibialis anterior (TA) muscle tissues were fixed in 10% formalin overnight, embedded in an optimal cutting temperature (OCT) compound (Sakura Finetek, Torrance, CA, USA) and immediately frozen in dry ice-cooled isopentane. Muscles in OCT were cut into 8-μm-thick cryosections using a cryostat (Thermo Scientific, Rockford, IL, USA) maintained at − 20 °C. For immunofluorescence staining, after washing in PBS, the sections were blocked in 5% BSA and incubated with a primary rabbit anti-laminin antibody (Abcam) in 5% BSA at 4 °C overnight, followed by incubation with Alexa Fluor® 647-conjugated secondary antibody (Life Technologies Corp., Carlsbad, CA, USA) for 1 h. Sections were further rinsed in PBS and treated with 4,6-diamidino-2-phenylindole (DAPI) for staining nuclei. Slides were visualized with a Nikon ECLIPSE Ti-U inverted microscope and Nikon camera using NIS-Elements software. Muscle cross-sectional area, fibre typing, localization of nuclei within the muscle fibre and stem cells were used to assess muscle physiology.

### Preparation of bone marrow cells and flow cytometry

Total bone marrow (BM) cells were isolated from mouse femurs and tibias by grinding the tissues in RPMI 1640 medium (WelGENE, Gyeongsan-si, South Korea) containing 2% fetal bovine serum (FBS). Red blood cells (RBCs) were lysed in the presence or absence of ACK buffer (150 mM NH_4_Cl, 1.0 mM KHCO_3_ and 0.1 mM EDTA [pH 7.4]), and filtered through a strainer. Antibodies were purchased from BD Biosciences or BioLegend. BM cells were stained as previously described [31] and analysed using FACSCanto II (BD Biosciences, Qume Drive San Jose, CA, USA). For peripheral blood analysis, cells were washed with PBS, and RBCs were lysed using RBC lysis buffer (BioLegend, San Diego, CA, USA). Fc receptors were blocked with an unlabelled CD16/32 antibody (clone 93; BioLegend, San Diego, CA, USA). The cells were washed, and extracellular marker proteins were stained for 30 min at 4 °C with fluorophore-conjugated antibodies specific for CD45 (clone 30-F11), CD11b (clone M1/70), Ly6G (clone 1A8), Ly6C (clone AL-21) and CD3ε (clone 145-2C11) from BD Pharmingen, and CD45R/B220 (clone RA3-6B2) from BioLegend. The cells were washed twice and analysed using a Gallios™ flow cytometer (Beckman Coulter, CA, USA). Data were analysed using the FlowJo software (Biosciences, Franklin Lakes, NJ, USA).

### Separation of bone niche, lineage-negative cells and lineage-positive cells

BM cells were removed from the femur by flushing with 10 mL of ice-cold HBSS buffer, and BM-removed bones were frozen at − 80 °C until use. The lineage-negative and -positive cells were isolated from flushed total BM cells using the Direct Lineage Cell Depletion Kit (Miltenyi Biotec, Bergisch Gladbach, Germany) according to the manufacturer’s recommendations. Briefly, RBCs were lysed using RBC lysis buffer (BioLegend, San Diego, CA, USA). After washing with HBSS buffer (300*g* for 5 min at 4 °C), the cells were resuspended in PBS containing 0.5% BSA and 2 mM EDTA. The cells were then incubated for 10 min at 4 °C with a microbead-conjugated monoclonal anti-mouse CD5, CD45R/B220, CD11b, Gr-1 (Ly-6G/C), 7-4 and Ter-119 antibody cocktail. Antibody-labeled cells were passed through the midi-magnetic assisted cell sorter (MACS) selection column. Lineage-negative cell fractions from the filtrate were collected and enumerated to determine the number of viable cells. Similarly, retained cells in the column, which represent the magnetically labeled lineage-positive cells, were eluted outside of the magnetic field and were counted to determine cell viability. Isolated cells were frozen at − 80 °C until use.

### Statistical analysis

Data are expressed as the mean ± standard error of the mean (SEM). For alpha and beta diversities, Kruskal-Wallis statistics and pairwise permutational analysis of variance (PERMANOVA) statistics were calculated using QIIME2 [20], respectively. Comparisons between multiple groups were performed using the Tukey-Kramer HSD test after one-way analysis of variance (ANOVA). Other statistical tests (Student’s *t* test, Wilcoxon signed rank test and Wilcoxon-Mann-Whitney test) were performed using the GraphPad Prism v8 software (GraphPad, San Diego, CA, USA).

## Results

### Ageing-associated changes in compositional structure of gut microbiome

**Fig. 1:**
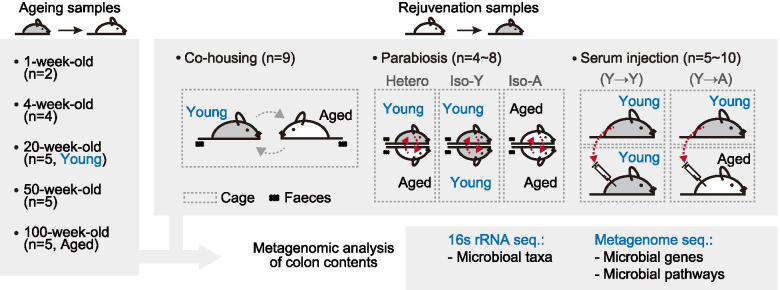
Overview of study samples. We collected gut microbial and host data from colon contents, intestinal biopsies, and blood samples in mice. For ageing experiments, we collected and profiled colon contents, intestinal biopsies, and blood samples from 1-, 4-, 20-, 50- and 100-week-old groups. For rejuvenation experiments, colon contents, intestinal biopsies and blood samples were collected from young (20-week-old groups) and aged mice (100-week-old groups) and applied to the three rejuvenation models, respectively. Co-housing and parabiosis experiments were performed for 6 weeks. For serum injection experiments, young and aged mice were systemically treated with serum isolated from young mice injected intravenously into the tail vein 8 times (for 3 weeks) or 16 times (for 6 weeks). To evaluate the ageing and rejuvenation efficacies, all mice were sacrificed at the end of the experiment. Metagenomic DNA was extracted from the collected colon contents, and 16S rRNA sequencing and metagenome shotgun sequencing were performed (Table S1)

We generated ageing and three rejuvenation models of C57BL/6J mice. Gut microbial and host data were collected from colon contents, intestinal biopsies, and blood samples of the ageing and rejuvenation models (Fig. 1). Each colon content obtained from all ageing and rejuvenation samples (*n* = 88) was subjected to 16S rRNA gene sequencing and shotgun metagenome sequencing, followed by profiling of microbial taxonomic composition and functional implications (Table S1).

**Fig. 2:**
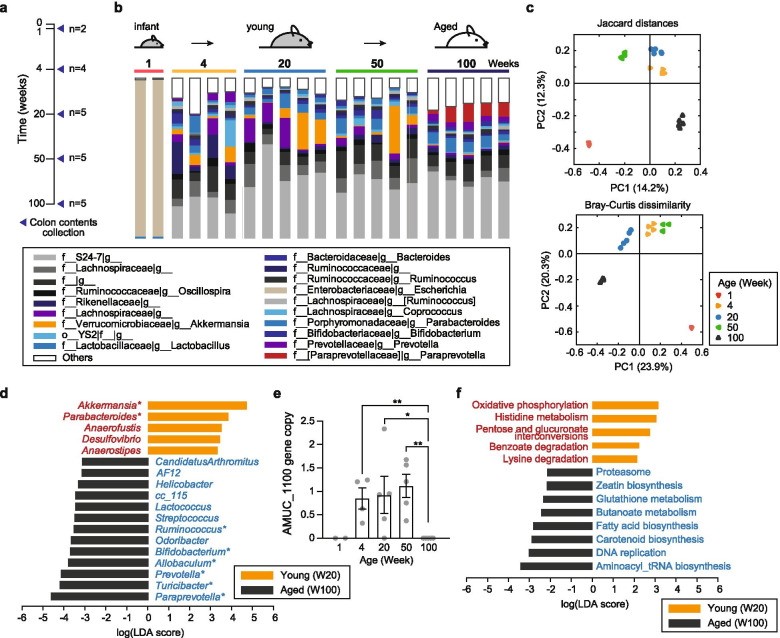
Metagenomic changes during the ageing process. **a** Schema of colon content collection during the ageing process. **b** Taxonomic composition for colon microbiota composition at the genus level, as determined with 16S rRNA sequencing. For clarity, the bacterial taxa with average abundance > 1% at each time point are shown. The taxonomic level is indicated as follows: ‘f’, family; ‘g’, genus. The unspecified ‘f__’ or ‘g__’ refer to OTUs without a specific family or genus name, respectively. **c** Principal coordinate analysis (PCoA) of β-diversity-based Jaccard distances (left panel) and Bray–Curtis dissimilarity metric (right panel) across five ageing groups (*P* = 0.001; permutational multivariate analysis of variance, PERMANOVA). Week-100 (W100, mice exhibit an aged phenotype) samples were significant different from week-4 (W4), week-20 (W20, mice exhibit a young phenotype) and week-50 (W50) samples (Table S2). **d** Linear discriminant analysis effect size (LEfSe) showing the taxonomic differences between young and aged mice at the genus level. Logarithmic linear discriminant analysis (LDA) score represents effect size associated with a bacterial taxon. Red and blue indicate significantly increased bacterial taxa in young mice (W20) and aged mice (W100), respectively. The asterisk indicates the bacterial taxa with average abundance > 1% at each time point. Threshold on the logarithmic LDA score was 2.0, and Kruskal-Wallis test *P* < 0.05. **e** Comparison of the AMUC_1100 gene abundance across five ageing groups. The copy number of AMUC_1100 was normalised with respect to the total number of genes in each metagenome (counts of AMUC_1100 / total number of ORFs × 1,000,000). Each value is the mean of biological replicate experiments, and error bars indicate ± SEM. Significance was assessed using the Mann-Whitney *U* test (*P < 0.05; **P < 0.01). **f** LEfSe identified the differentially abundant KEGG pathway between young and aged mice. Young- and aged-enriched pathways are indicated with a positive logarithmic LDA score (blue) and negative logarithmic LDA score (black), respectively. Threshold on the logarithmic LDA score was 2.0, and the Kruskal-Wallis test *P* < 0.05. The KEGG modules of oxidative phosphorylation (ko00190), histidine metabolism (ko00340), pentose and glucuronate interconversions (ko00040), benzoate degradation (ko00362), and lysine degradation (ko00310) were significantly (*P* < 0.01) abundant in the young groups, while those of carotenoid biosynthesis (ko00906), fatty acid biosynthesis (ko00061), butanoate metabolism (ko00650), glutathione metabolism (ko00480) and zeatin biosynthesis (ko00908) were significantly abundant in the aged mouse groups. Other results of LEfSe of metabolic pathways, comparing the 1-, 4- and 50-week-old groups to the 100-week-old group, are shown in Fig. S4a

First, we compared the gut microbiome of C57BL/6J mice at week 1 (W1), 4 (W4), 20 (W20), 50 (W50) and 100 (W100) to determine the differences in the gut microbiome composition according to ageing (Fig. 2a, b). Note that W20 and W100 exhibit a young and an aged phenotype, respectively. Compared to young mice, the Shannon and OTUs indices of bacterial communities in aged mice (W100) were significantly increased (PERMANOVA; *P* < 0.05), while the Faith’s phylogenetic diversity and Pielou’s evenness of bacterial communities in aged mice showed no differences (Fig. S1). Upon visualizing β-diversity (Fig. 2c and Fig. S2) with PERMANOVA of principal coordinate analysis (PCoA), we identified a differential clustering of W1, W4, W20, W50 and W100. In particular, the β-diversity between W20 and W100 was significantly different in all β-diversity indices (PERMANOVA, *P* < 0.01), suggesting age-dependent changes in their compositional structure of gut microbiome (Table S2).

To further corroborate these findings, we conducted LEfSe [22], representing the discriminatory taxa of gut microbiota at different ages. We observed that eight bacterial taxa were mainly dominated (average abundance > 1%) (Fig. 2d and Table S3). In young mice, young-specific bacterial taxa, such as *Akkermansia* (9.2% in average relative abundance) and *Parabacteroides* (2.2%), were highly abundant in the gut microbiota, whereas the relative abundance of *Akkermansia* (0.4%) and *Parabacteroides* (0.4%) decreased in aged mice. In contrast, the relative abundance of aged-specific bacterial taxa was more than 1.7-fold higher in aged mice than in young mice. While analysing the abundance of bacterial taxa in whole-genome shotgun metagenomic data (Fig. S3a), we also found that the most significant differences were a reduction in the abundance of *Akkermansia* in aged mice, together with an increase in *Turicibacter* and *Helicobacter* abundance (Fig. S3b). As expected, with an increase in the taxon level of *Akkermansia*, AMUC_1100 was significantly abundant (Mann-Whitney test *P* < 0.047) in the young mice (Fig. 2e) [11].

### Ageing-associated changes in gut microbial functions

To further understand the functional implications underlying the microbial differences between young and aged mice, we annotated the functions of enzymes identified in the whole-metagenome data. Total 10 KEGG metabolic pathways were significantly different between the young and aged groups (logarithmic LDA score > 2.0, *P* < 0.01) (Fig. 2f and Fig. S4a). Among them, three pathways (lysine degradation, fatty acid biosynthesis, butanoate metabolism) were found to be involved in the biosynthesis of SCFAs such as butyrate, and two pathways (histidine metabolism and glutathione metabolism) involved in γ-aminobutyric acid (GABA) biosynthesis were significantly enriched in young mice compared to aged mice. For more information about the ageing-associated changes in these pathways, please see note S1 and Fig. S4 in additional file 1. These findings clearly indicate that a significant microbiome shift occurs, and the specific functional potential related to butyrate and GABA biosynthesis significantly changes in response to host ageing.

### Rejuvenation procedure restores age-dependent alteration of intestinal function and inflammation

With the analysis of changes in the microbiome associated with ageing, we explored whether rejuvenating effects could be achieved in aged mice. For this, we remodelled the gut microbiota of the aged mice by co-housing [32], parabiosis (Fig. S5a) and serum injection [33]. We analysed several blood parameters related to liver, kidney, and muscle function as well as lipids (Table S4); however, we observed no significant changes in plasma markers by parabiotic pairing.

**Fig. 3:**
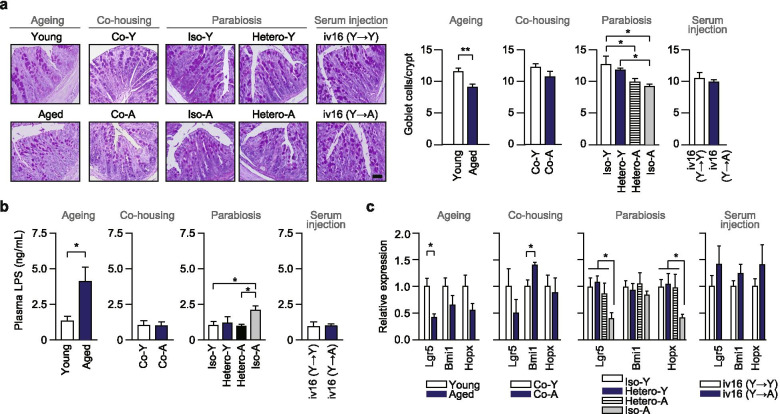
Rejuvenation procedure restores age-dependent alteration of intestinal function and inflammation. **a** Representative PAS-stained pictures (left panel) and mucus-containing goblet cell density in the colon (right panel), Scale bar 50 μm. **b** Circulating plasma LPS levels measured after 6 weeks of each experiment. **c** Gene expression of intestinal stem cell markers in the colon. All data are presented as means ± SEMs (*P < 0.05, two-tailed Student’s *t* test). *Co*-*Y* young mice from co-housing experiments, *Co*-*A* aged mice from co-housing experiments, *Hetero*-*Y* young mice from heterochronic pairs, *Hetero*-*A* aged mice from heterochronic pairs, *Iso*-*Y* young mice from isochronic young pairs, *Iso*-*A* aged mice from isochronic aged pairs, *iv 16* (Y→Y) young mice treated with serum isolated from young mice by intravenous injection into the tail vein 16 times, *iv 16* (Y→A) aged mice treated with serum isolated from young mice by intravenous injection into the tail vein 16 times

Several studies in rodents have reported an increase in intestinal permeability to macromolecules with age [34–36], indicating an age-associated decline in intestinal barrier function. To examine whether age-dependent differences in intestinal integrity and permeability could be altered by our rejuvenation protocols, we assessed the effects of co-housing, parabiotic pairing, and young serum injection on colonic histology, expression of genes associated with the gut barrier and plasma lipopolysaccharide (LPS) levels. Periodic acid Schiff’s (PAS)-positive goblet cell numbers in the colonic crypts were lower in aged mice than in young mice (Fig. 3a). Importantly, the number of PAS-positive goblet cells was not significantly different between rejuvenated mice and their counterparts during co-housing, parabiotic pairing, and serum injection. In the parabiosis experiment, we observed that the high expression of genes encoding secretory mucin proteins (Muc2 and Muc3) and the significant decrease in genes encoding barrier-forming tight junction proteins (Zo-1, Cldn3 and Cldn4) in isochronic aged mice (Fig. S5b and S5c). However, the expression of these genes in aged mice from the heterochronic group was restored to a similar degree as that observed in isochronic young mice.

Next, we measured the levels of circulating LPS as a marker of systemic inflammation by an age-dependent increase in intestinal permeability. Plasma LPS levels of naïve aged mice showed large intergroup variation; the LPS levels were approximately 3-fold higher than those of naïve young mice (Fig. 3b). Co-housing of aged mice with young mice and injection of young serum to aged mice normalised the blood LPS concentration in aged mice to the same extent as in young mice. Surprisingly, plasma LPS levels were significantly increased after parabiotic surgery and were approximately 2-fold higher in aged mice (iso-aged) than in young mice (iso-young). However, their aged counterparts from the hetero-aged group showed significantly decreased circulating LPS levels compared to the aged mice from the iso-aged group (Fig. 3b), indicating that the age-related gut inflammation was improved by rejuvenation, including sharing of young blood.

To investigate the effects of rejuvenation on intestinal homeostasis, we evaluated the expression of the intestinal stem cell (ISC) markers, in each colon sample from one ageing model and three experimental rejuvenation models (Fig. 3c). Aged mice showed a significant decrease in colonic Lgr5 (CBC cell marker) expression compared to young mice. However, the expression of Bmi1 was significantly increased in aged mice from the co-housing experiment and no significant changes in colonic ISC gene expression between young and aged mice in the serum injection model. In the parabiosis experiment, the expression of both Lgr5 and Hopx was remarkably downregulated in the colon of isochronic aged pairs compared to the isochronic young pairs, and heterochronic parabiosis led to a significant upregulation of these genes in aged mice (fold-change > 2). Further, we observed parabiosis experiments restore the expression of canonical Wnt signalling target genes and genes regulating ISC function (please see Note S2 and Fig. S5d–5i in additional file 1). These data suggest that the improvement of intestinal function and reduction of inflammation in aged mice might be attributed to rejuvenation.

### Changes in ageing-associated microbial signature during rejuvenation

**Fig. 4:**
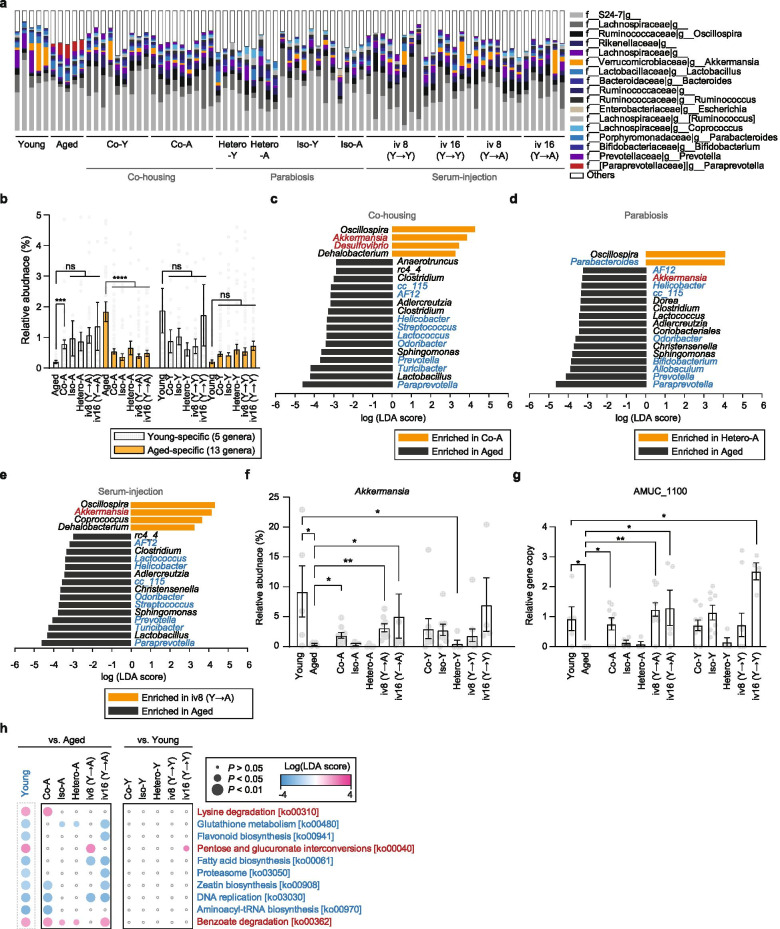
Several rejuvenation experiments induce changes in global microbial patterns. **a** Dynamics of dominant families and genera of colon microbiota composition during the rejuvenation procedure. For clarity, the bacterial taxa with an average abundance > 1% at each time point are shown. The taxonomic level is indicated as follows: ‘f’, family; ‘g’, genus. The unspecified ‘f__’ or ‘g__’ refer to OTUs without a specific family or genus name, respectively. **b** The relative abundance of the key prevalent microbial genera in the samples of aged mice during each rejuvenation procedure. ‘Young-specific’ indicates the increased abundance of bacterial taxa, such as *Akkermansia*, *Parabacteroides*, *Anaerofustis*, *Desulfovibrio* and *Anaerostipes*, in young mice (W20). ‘Aged-specific’ bacterial taxon indicates the increased abundance of bacterial taxa, such as *Candidatus Arthromitus*, AF12, *Helicobacter*, cc_115, *Lactococcus*, *Streptococcus*, *Ruminococcus*, *Odoribacter*, *Bifidobacterium*, *Allobaculum*, *Prevotella*, *Turicibacter* and *Paraprevotella*, in aged mice (W100). All data are means ± SEMs (Mann-Whitney *U* test; *P < 0.05; **P < 0.01; ***P < 0.001; ****P < 0.0001). **c–e** Logarithmic linear discriminant analysis (LDA) scores showing microbial genera that were significantly different in abundance between aged mice (W100) and **c** aged mice in the co-housing model, **d** aged mice in the parabiosis model or **e** aged mice in the serum injection model. Other pairwise LEfSe analyses are shown in Fig. S8. The blue and red genus names indicate enriched bacterial taxa in young mice (W20) and aged mice (W100), respectively. The threshold on the logarithmic LDA score was 2.0, and the Kruskal-Wallis test *P* < 0.05. **f** Relative abundance of the genus *Akkermansia* in colon microbiota during each rejuvenation procedure. All data are presented as means ± SEMs (Mann-Whitney *U* test; *P < 0.05; **P < 0.01). Each dot represents an individual mouse. **g** Comparison of the copy number of the AMUC_1100 gene in colon microbiota during each rejuvenation procedure. The copy number of AMUC_1100 was normalised with respect to the total number of genes in each metagenome (counts of AMUC_1100 / total number of ORFs × 1,000,000). All data are presented as the means ± SEMs (Mann-Whitney *U* test; *P < 0.05; **P < 0.01). Each dot represents an individual mouse. **h** LEfSe of metabolic pathways in the colon microbiota during each rejuvenation procedure. The two columns on the left indicate logarithmic LDA scores categorised by KEGG pathways, comparing the young group, Co-O, Iso-A, Hetero-A, iv 8 (Y→A) and iv 16 (Y→A) to the aged group. The last column on the right shows logarithmic LDA scores categorised by KEGG pathways, comparing Co-Y, Iso-Y, Hetero-Y, iv 8 (Y→Y) and iv 16 (Y→Y) to the young group. Only KEGG pathways that differed significantly between the young and aged groups are shown. Red and blue pathways indicate enriched metabolic pathways in young and aged mice, respectively. All KEGG pathways that differed significantly during the rejuvenation process are shown in Fig. S10. *Co*-*Y* young mice from co-housing experiments, *Co*-*A* aged mice from co-housing experiments, *Hetero*-*Y* young mice from heterochronic pairs, *Hetero*-*A* aged mice from heterochronic pairs, *Iso*-*Y* young mice from isochronic young pairs, *Iso*-*A* aged mice from isochronic aged pairs, *iv 8* (Y→Y) young mice treated with serum isolated from young mice by intravenous injection into the tail vein 8 times, *iv 16* (Y→Y) young mice treated with serum isolated from young mice by intravenous injection into the tail vein 16 times, *iv 8* (Y→A) aged mice treated with serum isolated from young mice by intravenous injection into the tail vein 8 times, *iv 16* (Y→A) aged mice treated with serum isolated from young mice by intravenous injection into the tail vein 16 times

To determine differences in the gut microbiome composition according to the rejuvenation process, we compared the composition of the gut microbiota of aged mice with that of the three rejuvenation models (Fig. 4a and Table S5). Analysis of α-diversity revealed that the rejuvenation process was associated with a trend for reduced bacterial diversity (Fig. S6). Analysis of taxonomic differences (Fig. 4a) and β-diversity analysis (Fig. S7a and S7b) revealed dynamic changes between aged mice and the three rejuvenation models.

We next checked whether the abundance of young- and aged-specific bacteria was associated with rejuvenation changes. Interestingly, the co-housing procedure significantly increased the relative abundance of young-specific bacteria (fold-change > 3.39, Mann-Whitney *U* test *P* < 0.0009) (Fig. 4b and Fig. S7c). In contrast, the relative abundance of aged-specific bacteria was significantly reduced during the rejuvenation procedure (fold-change < 0.25, Mann-Whitney *U* test, *P* < 0.001). Furthermore, LEfSe demonstrated that all rejuvenated mice were characterised by a significant increase in the abundance of *Oscillospira* and a significant reduction in the abundance of aged-specific genera, such as *Paraprevotella*, *Prevotella*, *Odoribacter*, *cc_115*, *AF12* and *Helicobacter* (Fig. 4c-e). Notably, it was predicted that *Oscillospira* could produce butyrate [37]. The abundance of the genus *Akkermansia* was significantly increased in rejuvenated mice during co-housing and serum injection (Mann-Whitney *U* test, *P* < 0.02), while the abundance of *Akkermansia* was significantly reduced in young mice during parabiosis (Mann-Whitney *U* test, *P* < 0.03) (Fig. 4f and Fig. S8). These results are consistent with those of a previous study showing that stress-induced corticosterone levels are negatively correlated with the relative abundance of *Akkermansia* in the gut (Fig. S9) [38]. Overall, our results suggest that all rejuvenation procedures can alter microbial communities.

Metagenomic analysis of the gut microbiota of the three rejuvenation models was conducted to further elucidate the rejuvenation-associated changes in gut microbial function. The data revealed the most significant differences in the abundance of *Akkermansia* (fold-change > 2.30, *q* value < 0.0004) in rejuvenated mice of the co-housing and serum injection (iv 8, Y→A) experiments, as well as a reduction in the abundance of *Turicibacter* (fold-change < 0.48, *q* value < 0.0004) and *Helicobacter* (fold-change < 0.32, *q* value < 1.25 × 10^−10^) in rejuvenated mice of co-housing, parabiosis (*Helicobacter* only), and serum injection experiments, corroborating the 16S rRNA gene sequencing results (Fig. S10). With an increase in the level of *Akkermansia*, the abundance of AMUC_1100 was also significantly increased in Co-A, iv8 (Y→A) and iv16 (Y→A) (Mann-Whitney *U* test, *P* < 0.03), indicating that co-housing and serum injection stimulated the growth of *Akkermansia* in the intestine of aged mice (Fig. 4g).

Remarkably, rejuvenated mice have enriched metabolic pathways similar to young mice, when compared to aged mice; however, there was no significant change, compared to that before rejuvenation, in the young mice from the rejuvenation pair (Fig. 4h and Fig. S11a). More specifically, the abundance of key enzymes related to pentose and glucuronate interconversions, lysine degradation, fatty acid biosynthesis and glutathione metabolism in rejuvenated mice changed to a level similar to that in young mice after rejuvenation (Fig. S11b–e). Among these, the *fabB* gene of fatty acid biosynthesis (fold-change < 0.22, *q* value < 1.27 × 10^−10^) and glutamate-cysteine ligase of glutathione metabolism (fold-change < 0.48, *q* value < 0.042) showed the most prominent change in all rejuvenated groups, resulting in the possibility of increased accumulation of glutamate and butyryl-CoA. Thus, the three rejuvenation procedure experiments converted the gut microbial consortia of aged mice, similar to that of the young-specific gut microbiota, thereby increasing the abundance of genes related to the butyrate and GABA biosynthesis pathways.

### Administration of Akkermansia muciniphila improves intestinal integrity and homeostasis

**Fig. 5:**
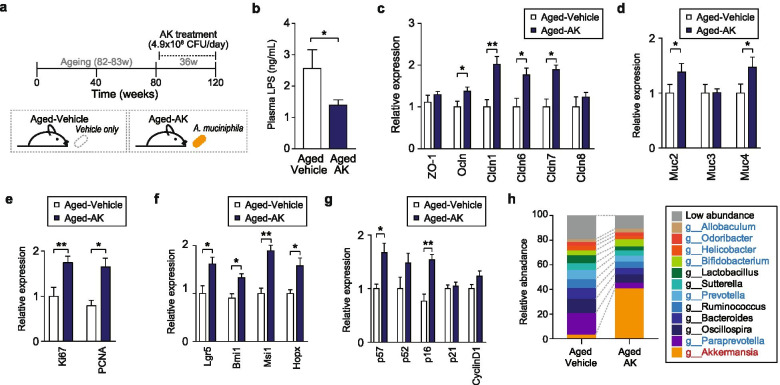
Administration of *Akkermansia muciniphila* (AK) improves intestinal integrity and the haematopoietic phenotype in aged mice. **a** Experimental scheme for oral administration of AK or vehicle only. AK was orally administered for 36 weeks. To evaluate the rejuvenation efficacy, all mice were sacrificed at the end of the experiment. Aged-vehicle, *n* = 10; aged-AK, *n* = 11. **b** Circulating plasma LPS levels in AK-treated and untreated aged mice. Data are means ± SEMs (*P < 0.05, two-tailed Student’s *t* test). **c–g** Gene expression of **c** barrier-forming tight junction proteins, **d** mucin, **e** cell proliferation, **f** intestinal stem cell and **g** cell cycle markers in the colon of AK-treated and untreated aged mice. Data are presented as means ± SEMs (*P < 0.05, two-tailed Student’s *t* test). **h** Comparison of taxonomic composition, at the genus level, between AK-treated and untreated aged mice. For clarity, the bacterial taxa with an average abundance > 1% at each time point are shown. Blue and red indicate enriched bacterial taxa in young and aged mice, respectively

Based on the gut metagenome analysis, we noted that *Akkermansia* is a major bacterium that is significantly involved in the rejuvenation of intestinal integrity and the immune system. To validate its biological importance identified in the metagenomic analysis, 82–83-week-old mice were subjected to gavage with AK pellets (4.9 × 10^8^ CFU/day) (Fig. 5a). To confirm the intestinal barrier function between the untreated group (aged-vehicle) and the AK-treated group (aged-AK), the levels of LPS in the blood were measured (Fig. 5b). The level of LPS in the AK-treated group was lower (two-tailed Student’s *t* test, *P* < 0.05) than that in the untreated group. The increased expression level of genes of tight junction proteins (Ocln, Cldn1, Cldn 6 and Cldn 7) and secretory mucin proteins (Muc2 and Muc4) in AK-treated group demonstrate that AK alleviated the ageing-induced weakened intestinal barrier function and inhibited the infiltration of pro-inflammatory molecules, such as LPS, by preserving the intestinal barrier function (Fig. 5c, d).

Next, mRNA levels of proliferation and ISC markers were compared to evaluate intestinal homeostasis. In the AK-treated group, the levels of the proliferation markers Ki67 and PCNA were significantly increased (Fig. 5e). Moreover, the levels of the ISC markers Lgr5, Bmi1, Msi1 and Hopx were significantly higher in the AK-treated group than in the untreated group (Fig. 5f). Among the cell cycle regulator genes, the mRNA expression of p27, p16 and cyclin D1 was significantly increased in the AK-treated group (Fig. 5g), indicating increased proliferation and regeneration of intestinal cells. Furthermore, administration of AK restores the expression of the canonical Wnt signalling target genes and ameliorates the senescence-related phenotype in haematopoietic system by controlling ICAM-1 expression, particularly in the BM niche of mice (please see Note S3 and Fig. S12 in additional file 1). These results demonstrate that oral administration of AK improves impaired intestinal homeostasis due to ageing by accelerating the renewal and turnover of intestinal cells.

Next, we performed 16S and metagenome sequencing of colon samples collected from AK-treated and -untreated mice. Although the two groups did not vary in terms of α-diversity (Table S2), the β-diversity analysis demonstrated distinct microbial communities (PERMANOVA, *P* < 0.01) between the two groups (Table S2 and Fig. S13a). Compared to the AK-untreated group, the AK-treated group had a significantly higher abundance of AK in their gut microbiome, as determined by 16S rRNA sequencing (fold-change = 11.8, Mann-Whitney test, *P* < 0.0001) (Table S6) and metagenome sequencing data (fold-change = 9.78, *q* value = 7.34 × 10^−24^) (Fig. 5h and Fig. S13b). Additionally, LEfSe indicated a significant increase in the abundance of AK and *Allobaculum* in AK-treated mice (Kruskal-Wallis test, *P* = 0.0002) (Fig. S13c), while abundance of *Paraprevotella*, which is an aged-specific genus, was significantly reduced (Kruskal-Wallis test, *P* = 0.006). Of note, *Allobaculum* is related to SCFA production in the gut microbiota [39]. Furthermore, metabolic pathway analysis revealed that only four proteins were highly abundant (Kruskal-Wallis test, *P* < 0.05, logarithmic LDA 2.0) in the two groups (Fig. S13d), and no metabolic pathways were enriched between the two groups. Taken together, these results demonstrate that the intestinal ageing phenotypes of aged mice could be alleviated by oral administration of AK without altering the specific metabolic pathway of the intestinal microbiota.

### Oral administration of AK extends healthy lifespan

**Fig. 6:**
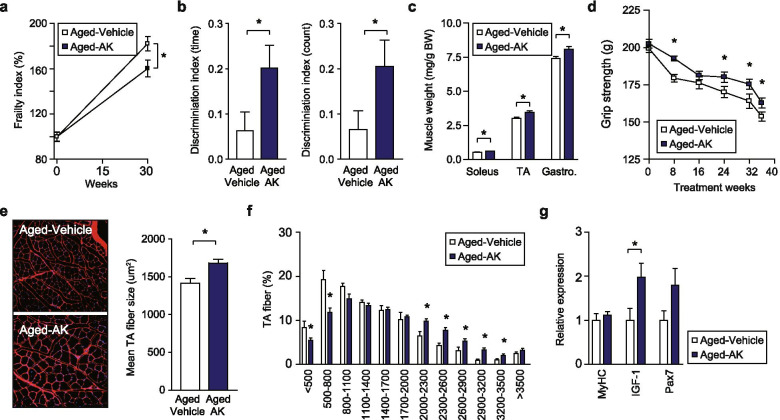
*Akkermansia muciniphila* (AK) treatment improves healthy lifespan. **a** Frailty index and **b** discrimination index of AK-treated and untreated aged mice. **c** Weight of soleus, tibialis anterior (TA) and gastrocnemius (gastro) skeletal muscles. **d** Grip strength comparison between AK-treated and untreated aged mice. **e** Pictures are representative images of laminin-stained TA muscles (left panel). Mean TA fibre size (right panel). **f** Frequency distribution of cross-sectional TA fibre area. **g** qRT-PCR analysis of MyHC, *Igf-1* and *Pax7* mRNA expression in TA muscle of the aged mice treated with AK. Data are presented as means ± SEMs (*P < 0.05, two-tailed Student’s test)

Finally, we investigated whether AK prolongs the lifespan and muscle function of aged mice. In contrast to the progeroid mice (lmnaG609G/G609G) [40], AK treatment did not affect the mean or maximum lifespan of C57BL/6J mice (Fig. S13e). In addition, AK treatment did not alter the average body weight (Fig. S13f) between the experimental groups. A gross necropsy examination of all mice that died revealed no histopathological differences between the groups, confirming that AK was nontoxic at the given doses. Interestingly, we observed that oral administration of AK led to an improvement in the FI (Table S7) and cognitive activity when aged mice were treated with AK (Fig. 6a, b). In the recognition session with two different objects (one novel and the other familiar), AK-untreated mice exhibited a significantly lower discrimination index (DI) than the AK-treated mice, which is consistent with impaired cognition. AK treatment markedly increased the DI in aged mice, reflecting the therapeutic effect of AK treatment on age-related phenotypes and cognitive impairment.

Additionally, we observed that AK-treated aged mice had better muscle strength, as assessed with a grip test and higher skeletal muscle mass (Fig. 6c, d), than the control mice. Examination of muscle fibre size distribution further revealed that AK-treated mice showed a significantly larger mean size of total calculated fibres (Fig. 6e) and a shift towards larger myofibers, as compared to AK-untreated mice (Fig. 6f). qRT-PCR data showed that AK treatment increased the expression of not only *Pax7* (muscle stem cells, not significant) but also *Igf-1* (*P* < 0.05), which is involved in the hypertrophy of aged muscle (Fig. 6g), thereby supporting the potential of AK as a new candidate therapeutic agent for sarcopenia. Thus, our data demonstrated that the high abundance of AK in the gut could extend the healthy lifespan, as evidenced by the FI, cognitive function and restoration of muscle atrophy.

## Discussion

Ageing-induced changes in the composition of the gut microbiota are associated with various age-related disorders. Also, the transfer of gut microbiota from aged mice to young germ-free mice suggested that ageing-related remodelling of the gut microbiota may contribute to inflammation [7, 41]. However, the rejuvenating effects of altered gut microbiota on the presence of specific bacteria remain elusive. Here, we show the changes in key microbial communities and their functions during ageing and three rejuvenation procedures, and the increase in the healthy lifespan of aged mice by oral administration of AK.

Our results indicate that intestinal function, inflammation and intestinal homeostasis of aged mice can be rescued by three rejuvenation intervention models. All rejuvenation procedures significantly increased the relative abundance of the butyrate producer *Oscillospira* in rejuvenated mice, while the abundance of the beneficial genus *Akkermansia* was significantly increased in rejuvenated mice only during co-housing and serum injection. In addition, we observed that the abundance of aged-specific genera, such as *Paraprevotella*, *Prevotella*, *Odoribacter*, *cc_115*, *AF12* and *Helicobacter*, was significantly decreased in all rejuvenated mice, suggesting that the relative abundance of young- and aged-specific bacteria was reversed in their young counterparts during the co-housing and parabiosis procedures. It has been reported that a high abundance of *Prevotella*, *Turicibacter* and *Paraprevotella* is associated with dysbiosis, chronic inflammation and type 2 diabetes [42–45], suggesting that they increase the risk of inflammation. Defective intestinal function and inflammation in aged mice can be improved by gut microbiota remodelling, suggesting a causal link between age-related changes in the gut microbiome and age-dependent morbidities.

In this study, we evaluated the alterations in community-wide microbial functions in rejuvenation and AK treatment experiments. We observed that 10 enriched KEGG pathways found in rejuvenated mice were similar to those in young mice when compared to those in aged mice (Fig. 4h). Among these, lysine degradation and pentose and glucuronate interconversions (pectin degradation pathway) are of particular interest because SCFAs such as butyrate, propionate and acetate are the major fermentation products derived from the gut microbial degradation of lysine [46, 47] and dietary fibre [8, 48] (Fig. S4h). The key enzyme of the *buk*-mediated pathway was also significantly more abundant in the young and rejuvenated groups than in the aged groups, while *ptb* and *buk* were less abundant in butanoate metabolism (Note S1 and Fig. S4). The high abundance of the *buk*-mediated route in the butanoate metabolism is consistent with the results of previous metagenome analysis [49].

Furthermore, we found that none of the specific metabolic pathways of the intestinal microbiota were significantly altered by oral administration of AK (Fig. S13d) in the AK experiment. In line with these results, a recent study showed that oral administration of pasteurised AK increases the intestinal concentrations of several anti-ageing metabolites, including bile acids, SCFAs, 2-hydroxybutyrate and polyamines [50]. These findings suggest that rather than AK directly producing the metabolites, AMUC_1100 protein or other factors derived from its pasteurised form act to regulate a gut condition where bacteria can produce beneficial metabolites indirectly [9, 11].

Additionally, we observed that the benzoate degradation pathway was significantly enriched in young and aged mice rejuvenated by the three rejuvenation methods (Fig. 4h). Benzoate is a well-known antibacterial agent [51], and a recent study revealed that its catabolic pathways exist in human gut metagenomic datasets [52], and antimicrobial food additives induce enrichment of their catabolic pathways at the metagenomic level [53]. Likewise, we may argue that rejuvenated mice are more resistant to benzoate than aged mice. However, to date, there have been no studies on the relationship between benzoate degradation and host ageing.

For effective anti-ageing, it is important to find an appropriate approach to specifically manipulate the microbiota. Although FMT has shown potential in the treatment of several diseases [32, 40], it is a complex biological intervention and has intrinsic limitations, that is, it can use only feces from healthy donors that are free from diseases. In this study, we controlled the ageing-related phenotype by oral administration of a single microbe, AK. AK restores intestinal integrity by activating epithelial cells, thereby supporting the growth of other beneficial commensals through a mechanism dependent on AMUC_1100 [11]. In line with previous studies, our results demonstrated that oral administration of AK improves impaired intestinal homeostasis by activating the Wnt/β-catenin signalling pathway and ameliorates senescence-related phenotypes in the haematopoietic system (Fig. S13g). Furthermore, our data shows that AK extends the healthy lifespan, as evidenced by the FI and restoration of muscle atrophy. Since the age-related inflammatory state is associated with a decrease in skeletal muscle size and function (sarcopenia) [54], the decrease in inflammation caused by oral administration of AK may be involved in the restoration of muscle metabolic function.

## Conclusions

Our work reveals the microbial composition and gene abundance in the gut microbiota changed dynamically during the ageing and rejuvenation experiments. In addition, oral administration of AK strains alleviated age-related symptoms, thereby increasing the healthspan. These findings could be helpful in developing therapeutic strategies to promote healthy, active ageing.

## Supplementary Information

**Additional file 1: Note S1.** Ageing-associated changes in microbial functional potential and metabolism. **Note S2.** Intestinal stem cell markers and de novo crypt formation in the colon of rejuvenated mice. **Note S3.** Administration of *Akkermansia muciniphila* improves canonical Wnt signalling and the senescence-related phenotype in the intestinal stem cell function and haematopoietic system. **Fig. S1.** Alpha-diversity and bacterial abundances at different ages. a–d, α-diversity based (a) Shannon, (b) observed OTU, (c) Faith's phylogenetic diversity, (d) evenness indices across the ageing process. Statistical testing showed a significant difference for Shannon diversity and observed species, while faith-PD richness was not significantly different in aged mice compared to young mice. Statistical analysis was performed using Kruskal-Wallis test (*, *P* < 0.05; **, *P* < 0.01). e–g, Heatmap for the bacteria abundances for the mice across ageing groups at the (e) order, (f) family, and (g) genus level. The taxonomic units with average abundance > 1% in each sampling time point are shown. **Fig. S2.** Beta-diversity at different ages. Principal coordinate analysis (PCoA) of β-diversity based weighted unifrac (left panel) and unweighted unifrac metric (right panel) across five ageing groups (P = 0.001; permutational multivariate analysis of variance, PERMANOVA). 100-week-old groups show significant differences with 4-week, 20-week, and 50-week-old groups (Table S2). **Fig. S3.** Taxonomic composition and difference during the ageing process determined by metagenomic sequencing. a, Taxonomic composition for colon microbiota composition determined by metagenomic sequencing at the genus level. The average relative abundance of the top 25 most abundant taxa in all samples is shown. b, A significant relative abundance change in response to ageing process. Bacterial taxon showing a significant abundance of change (q-value < 0.01) was only shown with average fold-change value at the genus level. Red and blue indicate significantly increased bacterial taxa in young mice (W20) of 16s rRNA sequencing data and aged mice (W100) of 16s rRNA sequencing data, respectively. The Benjamini and Hochberg's FDR control method was used to correct for multiple comparisons. **Fig. S4.** Ageing-associated changes in microbial functional potential and metabolism. a, LEfSe analysis of metabolic pathways in colon samples from young group compared to aged group are shown. The four columns on the left show logarithmic linear discriminant analysis (LDA) score of KEGG metabolic pathway, comparing the 1-week, 4-week, 20-week, and 50-week-aged groups to 100-week-old group. Red and blue pathway indicate enriched metabolic pathway in young mice and in aged mice, respectively. b–g, Enriched metabolic pathway presented in young mice compared to aged mice. Horizontal bar plot reflect the fold-change of each enzyme involved in (b) lysine degradation, (c) histidine metabolism, (d) pentose and glucuronate interconversions, (e) fatty acid biosynthesis, (f) glutathione metabolism, (g) and butanoate metabolism in 1-week, 4-week, 20-week, and 50-week-old groups compared to 100-week-old group. Red and blue KEGG reactions indicate high abundance in young mice and in aged mice, respectively. The following genes are represented by enzyme name and EC number: OADH, 2-oxoglutarate dehydrogenase (OADH) [EC:2.3.1.61]; DAT, D-alanine transaminase [EC:2.6.1.21]; glutaryl-CoA dehydrogenase [EC:1.3.8.6]; lysine 2,3-aminomutase [EC:5.4.3.2]; beta-lysine 5,6-aminomutase [EC:5.4.3.3]; trans-2-enoyl-CoA reductase [EC:1.3.1.38]; acyl-CoA thioesterase YciA [EC:3.1.2.-]; PRPP, phosphoribosyl pyrophosphate; phosphoribosyl-ATP pyrophosphohydrolase [EC:3.6.1.31]; phosphoribosyl-AMP cyclohydrolase [EC:3.5.4.19]; phosphoribosylformimino-5-aminoimidazole carboxamide ribotide isomerase [EC:5.3.1.16]; imidazoleglycerol-phosphate dehydratase [EC:4.2.1.19]; histidinol-phosphate aminotransferase [EC:2.6.1.9]; histidinol-phosphatase (PHP family) [EC:3.1.3.15]; histidinol dehydrogenase [EC:1.1.1.23]; histidine ammonia-lyase [EC:4.3.1.3]; urocanate hydratase [EC:4.2.1.49]; imidazolonepropionase [EC:3.5.2.7]; GAD, glutamate decarboxylase [EC:4.1.1.15]; pectinesterase [EC:3.1.1.11]; pectate lyase [EC:4.2.2.2]; pectate disaccharide-lyase [EC:4.2.2.9]; oligogalacturonide lyase [EC:4.2.2.6]; glucuronate isomerase [EC:5.3.1.12]; tagaturonate reductase [EC:1.1.1.58]; altronate hydrolase [EC:4.2.1.7]; mannonate dehydratase [EC:4.2.1.8]; fructuronate reductase [EC:1.1.1.57]; L-xylulokinase [EC:2.7.1.53]; 3-dehydro-L-gulonate-6-phosphate decarboxylase [EC:4.1.1.85]; L-xylulokinase [EC:2.7.1.53]; L-ribulokinase [EC:2.7.1.16]; L-arabinose isomerase [EC:5.3.1.4]; xylulokinase [EC:2.7.1.17]; L-xylulose reductase [EC:1.1.1.10]; aldehyde reductase [EC:1.1.1.21]; D-xylulose reductase [EC:1.1.1.9]; rhamnulokinase [EC:2.7.1.5]; rhamnulose-1-phosphate aldolase [EC:4.1.2.19]; FabD, [acyl-carrier-protein] S-malonyltransferase [EC:2.3.1.39]; FabH, 3-oxoacyl-[acyl-carrier-protein] synthase III [EC:2.3.1.180]; FabG, 3-oxoacyl-[acyl-carrier protein] reductase [EC:1.1.1.100]; FabZ, 3-hydroxyacyl-[acyl-carrier-protein] dehydratase [EC:4.2.1.59]; FabK, enoyl-[acyl-carrier protein] reductase II [EC:1.3.1.-]; FabL, enoyl-[acyl-carrier protein] reductase III [EC:1.3.1.-]; FabF, 3-oxoacyl-[acyl-carrier-protein] synthase II [EC:2.3.1.179]; FabB, 3-oxoacyl-[acyl-carrier-protein] synthase I [EC:2.3.1.41]; YciA, acyl-CoA thioesterase [EC:3.1.2.-]; GSR, glutathione reductase (NADPH) [EC:1.8.1.7]; phospholipid-hydroperoxide glutathione peroxidase [EC:1.11.1.12]; PepA, leucyl aminopeptidase [EC:3.4.11.1]; PepD; dipeptidase D [EC:3.4.13.-]; PepN; aminopeptidase N [EC:3.4.11.2]; glutamate--cysteine ligase [EC:6.3.2.2]; GAD, glutamate decarboxylase [EC:4.1.1.15]; 2-hydroxyglutarate dehydrogenase [EC:1.1.99.2]; glutaconate CoA-transferase [EC:2.8.3.12]; ptb; phosphate butyryltransferase [EC:2.3.1.19]; buk; butyrate kinase [EC:2.7.2.7]; trans-2-enoyl-CoA reductase [EC:1.3.1.38]; acyl-CoA thioesterase YciA [EC:3.1.2.-]. h, Schematic summary shows key metabolic differences between young and aged group based on relative gene abundance profiles. Host metabolism is influenced by γ-aminobutyric acid (GABA) neurotransmitter, affecting the brain (inducing satiety) GABA modulates inflammation. Fermentation of pectin by intestinal-specific bacteria produces butyrate. It affects host metabolism in several ways by acting on the G protein-coupled receptor (GPR) expressed by intestinal endocrine cells. Butyrate stimulates the release of glucagon-like peptide 1 (GLP-1) and peptide YY (PYY), affecting the pancreas (inducing insulin secretion) and brain (inducing satiety). Lipopolysaccharides (LPS) derived from the membrane of Gram-negative bacteria are pro-inflammatory compounds. AMUC_1100 derived from *Akkermansia muciniphila* improves intestinal barrier function by increasing goblet cell density and stimulating Toll-like receptor 2 (TLR2). AMUC_1100 exerts the beneficial effect on mucus layer regeneration, inflammation, and insulin sensitivity. Arrow heads indicate stimulation and bar heads indicate inhibition. **Fig. S5.** Parabiosis experiments restores intestinal function, canonical Wnt signalling target genes, and genes regulating ISC function. a, Verification of blood sharing between the parabiotic pairs using Evans blue. Representative photographs of the mice were taken at 0.5 and 24 h after the injection of Evans blue into the tail vein of one parabiont in a pair a week after surgery. The serum concentration of Evans blue in both mice in each pair was measured at 620 nm by spectrophotometry at 0, 0.5, 1, 24, and 48 h after injection. b, Gene expression profile of mucin in the colon. c, Gene expression profile of barrier-forming tight junction proteins in the colon. d–g, Quantitative real-time PCR analyses for expression of canonical Wnt signalling target genes and genes regulating ISC function in ageing, cage model, serum injection model and parabiosis model. Data are means ± SEMs (*, *P* < 0.05, two-tailed Student’s t-test). ab means not sharing a common letter are significantly different at *P* < 0.05. h, Representative Ki67-stained pictures. Scale bar, 25 μm. i, quantification of Ki-67 positive cells per crypt base columnar cell in the colon. Data are means ± SEM. (*, *P* < 0.05, two-tailed Student’s t-test). **Fig. S6.** Dynamics of α-diversity indices including (a) Shannon diversity, (b) observed OTU, (c) Faith's phylogenetic diversity, (d) Pielou's evenness indices among samples of the co-housing, parabiosis, and serum injection groups. Statistical analysis was performed using Kruskal-Wallis test, young (week 20) versus rejuvenated mice group (*, P < 0.05; **, P < 0.01); aged (W100) versus rejuvenated mice group (#, *P* < 0.05; ##, *P* < 0.01). Abbreviations: Co-Y, young mice from co-housing experiments; Co-A, aged mice from co-housing experiments; Hetero-Y, young mice from heterochronic pairs; Hetero-A, aged mice from heterochronic pairs; Iso-Y, young mice from isochronic young pairs; Iso-A, aged mice from isochronic aged pairs; iv 8 (Y→Y), young mice treated with serum isolated from young mice by intravenously into the tail vein 8 times; iv 16 (Y→Y), young mice treated with serum isolated from young mice by intravenously into the tail vein 16 times; iv 8 (Y→A), aged mice treated with serum isolated from young mice by intravenously into the tail vein 8 times; iv 16 (Y→A), aged mice treated with serum isolated from young mice by intravenously into the tail vein 16 times. **Fig. S7.** Gut microbiome alteration in several rejuvenation models. a–b, Principal coordinate analysis (PCoA) of β-diversity based (a) Jaccard distances, and (b) Bray-Curtis dissimilarity metric among samples of the co-housing (left), parabiosis (middle) and serum injection (right) groups of mice analysed (PERMANOVA, P = 0.001). Each dot represents an individual mouse. c, Heatmap presenting the relative abundance (%) of the key prevalent bacterial taxa found in young and aged-mice. The red and blue genus indicates enriched bacterial taxa in young mice (W20) and aged mice (W100), respectively. **Fig. S8.** LEfSe analysis showing microbial genus that was significantly different in abundance between (a) young mice (W20) and Co-Y, (b) young mice and Hetero-Y, (c) Hetero-Y mice and Iso-Y, (d) Hetero-A and Iso-A, (e) aged mice and iv 16 (Y→A), (f) young mice and iv 8 (Y→Y), and (g) young mice and iv 16 (Y→Y). Abbreviations: Co-Y, young mice from co-housing experiments; Hetero-Y, young mice from heterochronic pairs; Hetero-A, aged mice from heterochronic pairs; Iso-Y, young mice from isochronic young pairs; Iso-A, aged mice from isochronic aged pairs; iv 8 (Y→Y), young mice treated with serum isolated from young mice by intravenously into the tail vein 8 times; iv 16 (Y→Y), young mice treated with serum isolated from young mice by intravenously into the tail vein 16 times; iv 16 (Y→A), aged mice treated with serum isolated from young mice by intravenously into the tail vein 16 times. Abbreviations: Co-Y, young mice from co-housing experiments; Co-A, aged mice from co-housing experiments; Hetero-Y, young mice from heterochronic pairs; Hetero-A, aged mice from heterochronic pairs; Iso-Y, young mice from isochronic young pairs; Iso-A, aged mice from isochronic aged pairs; iv 8 (Y→Y), young mice treated with serum isolated from young mice by intravenously into the tail vein 8 times; iv 16 (Y→Y), young mice treated with serum isolated from young mice by intravenously into the tail vein 16 times; iv 8 (Y→A), aged mice treated with serum isolated from young mice by intravenously into the tail vein 8 times; iv 16 (Y→A), aged mice treated with serum isolated from young mice by intravenously into the tail vein 16 times. **Fig. S9.** Parabiosis experiments induce corticosterone levels in plasma. Plasma corticosterone levels in (a) naïve mice (young and aged mice) and (b) heterochronic parabiotic paired mice. All data are means ± SEMs (Mann-Whitney U test; *, *P* < 0.05). **Fig. S10.** Taxonomic composition and difference during rejuvenation process determined by metagenomic sequencing. Several microbial genera were significantly different in abundance between (a) Co-A and aged, (b) Co-Y and young, (c) Hetero-A and aged, (d) Hetero-A and Iso-A, (e) Hetero-Y and Young, (f) iv 8 (Y→A) and aged, (g) iv 16 (Y→A) and aged, (h) iv 8 (Y→Y) and young, and (i) iv 16 (Y→Y) and young. Bacterial taxon showing a significant abundance of change (q-value < 0.001) was only shown with average fold-change value at the genus level. Red and blue indicate significantly increased bacterial taxa in young mice of 16s rRNA sequencing data and mice of 16s rRNA sequencing data, respectively. The Benjamini and Hochberg's FDR control method was used to correct for multiple comparisons. Abbreviations: Co-Y, young mice from co-housing experiments; Co-A, aged mice from co-housing experiments; Hetero-Y, young mice from heterochronic pairs; Hetero-A, aged mice from heterochronic pairs; Iso-Y, young mice from isochronic young pairs; Iso-A, aged mice from isochronic aged pairs; iv 8 (Y→Y), young mice treated with serum isolated from young mice by intravenously into the tail vein 8 times; iv 16 (Y→Y), young mice treated with serum isolated from young mice by intravenously into the tail vein 16 times; iv 8 (Y→A), aged mice treated with serum isolated from young mice by intravenously into the tail vein 8 times; iv 16 (Y→A), aged mice treated with serum isolated from young mice by intravenously into the tail vein 16 times. **Fig. S11.** Rejuvenation-associated changes in microbial functional potential and metabolism. a, LEfSe analysis of metabolic pathways in colon microbiota during each rejuvenation procedure. The two columns on the left indicate logarithmic LDA scores categorised by KEGG pathways, comparing young group, Co-A, Iso-A, Hetero-A, iv 8 (Y→A), and iv 16 (Y→A) to the aged group. The last column on the right shows logarithmic LDA scores categorised by KEGG pathways, comparing Co-Y, Iso-Y, Hetero-Y, iv 8 (Y→Y), and iv 16 (Y→Y) to the young group. The red and blue names of pathways indicate enriched metabolic pathways in young mice and in aged mice, respectively. Only KEGG pathways differ significantly during each rejuvenation procedure. b–e, Shown horizontal bar plots indicate the fold-change of each enzyme involved in (b) pentose and glucuronate interconversions, (c) lysine degradation, (d) glutathione metabolism, and (e) fatty acid biosynthesis in young, Co-A, Hetero-A, iv8 (Y→A), iv16 (Y→A), Co-Y, Hetero-Y, iv8 (Y→Y), and iv16 (Y→Y). Red and blue KEGG reactions indicate high abundance in young mice and in aged mice, respectively. Abbreviations: Co-Y, young mice from co-housing experiments; Hetero-Y, young mice from heterochronic pairs; Hetero-A, aged mice from heterochronic pairs; Iso-Y, young mice from isochronic young pairs; Iso-A, aged mice from isochronic aged pairs; iv 8 (Y→Y), young mice treated with serum isolated from young mice by intravenously into the tail vein 8 times; iv 16 (Y→Y), young mice treated with serum isolated from young mice by intravenously into the tail vein 16 times; iv 16 (Y→A), aged mice treated with serum isolated from young mice by intravenously into the tail vein 16 times. The following genes are represented by enzyme name and EC number: OADH, 2-oxoglutarate dehydrogenase (OADH) [EC:2.3.1.61]; DAT, D-alanine transaminase [EC:2.6.1.21]; glutaryl-CoA dehydrogenase [EC:1.3.8.6]; lysine 2,3-aminomutase [EC:5.4.3.2]; beta-lysine 5,6-aminomutase [EC:5.4.3.3]; trans-2-enoyl-CoA reductase [EC:1.3.1.38]; acyl-CoA thioesterase YciA [EC:3.1.2.-]; GAD, glutamate decarboxylase [EC:4.1.1.15]; pectinesterase [EC:3.1.1.11]; pectate lyase [EC:4.2.2.2]; pectate disaccharide-lyase [EC:4.2.2.9]; oligogalacturonide lyase [EC:4.2.2.6]; glucuronate isomerase [EC:5.3.1.12]; tagaturonate reductase [EC:1.1.1.58]; altronate hydrolase [EC:4.2.1.7]; mannonate dehydratase [EC:4.2.1.8]; fructuronate reductase [EC:1.1.1.57]; L-xylulokinase [EC:2.7.1.53]; 3-dehydro-L-gulonate-6-phosphate decarboxylase [EC:4.1.1.85]; L-xylulokinase [EC:2.7.1.53]; L-ribulokinase [EC:2.7.1.16]; L-arabinose isomerase [EC:5.3.1.4]; xylulokinase [EC:2.7.1.17]; L-xylulose reductase [EC:1.1.1.10]; aldehyde reductase [EC:1.1.1.21]; D-xylulose reductase [EC:1.1.1.9]; rhamnulokinase [EC:2.7.1.5]; rhamnulose-1-phosphate aldolase [EC:4.1.2.19]; FabD, [acyl-carrier-protein] S-malonyltransferase [EC:2.3.1.39]; FabH, 3-oxoacyl-[acyl-carrier-protein] synthase III [EC:2.3.1.180]; FabG, 3-oxoacyl-[acyl-carrier protein] reductase [EC:1.1.1.100]; FabZ, 3-hydroxyacyl-[acyl-carrier-protein] dehydratase [EC:4.2.1.59]; FabK, enoyl-[acyl-carrier protein] reductase II [EC:1.3.1.-]; FabL, enoyl-[acyl-carrier protein] reductase III [EC:1.3.1.-]; FabF, 3-oxoacyl-[acyl-carrier-protein] synthase II [EC:2.3.1.179]; FabB, 3-oxoacyl-[acyl-carrier-protein] synthase I [EC:2.3.1.41]; YciA, acyl-CoA thioesterase [EC:3.1.2.-]; GSR, glutathione reductase (NADPH) [EC:1.8.1.7]; phospholipid-hydroperoxide glutathione peroxidase [EC:1.11.1.12]; PepA, leucyl aminopeptidase [EC:3.4.11.1]; PepD; dipeptidase D [EC:3.4.13.-]; PepN; aminopeptidase N [EC:3.4.11.2]; glutamate--cysteine ligase [EC:6.3.2.2]; GAD, glutamate decarboxylase [EC:4.1.1.15]; trans-2-enoyl-CoA reductase [EC:1.3.1.38]; acyl-CoA thioesterase YciA [EC:3.1.2.-]. **Fig. S12.** Administration of AK restores the expression of the canonical Wnt signalling target genes and ameliorates the senescence-related phenotype in haematopoietic system. a, Quantitative reverse transcription PCR (qRT-PCR) analyses for the expression of canonical Wnt signalling target genes and genes regulating ISC function. Data are presented as means ± SEMs (*, *P* < 0.05, two-tailed Student’s t-test). b, A representative fluorescence-activated cell sorting plot showing the frequencies of LT-HSCs, ST-HSCs, and MPPs among LSKs in the bone marrow of AK-treated and untreated aged mice. c, Percentages of LT-HSCs, ST-HSCs, and MPPs among LSKs. Data are presented means ± SEMs (*, *P* < 0.05, two-tailed Student’s t-test). d, Representative images and e, frequencies of neutrophils, T cells, and B cells in the blood of AK-treated and untreated aged mice. Data are presented as means ± SEMs (*, *P* < 0.05, two-tailed Student’s t-test). f, qRT-PCR analysis of the Icam1 mRNA purified from bone niches and lineage-negative and -positive cells of the aged mice treated with AK. Data are presented as means ± SEMs (*, *P* < 0.05, two-tailed Student’s t-test). **Fig. S13.** Effect of AK treatment in aged mice. a, Principal coordinate analysis (PCoA) of β-diversity based Jaccard distances and Bray-Curtis dissimilarity metric between AK-treated and untreated aged mice (*P* = 0.001; permutational multivariate analysis of variance, PERMANOVA). The gut microbiome of AK-treated aged mice shows significant differences with control (Table S2). b, Taxonomic composition and difference determined by metagenomic sequencing. Microbial genera were significantly different in abundance between aged-AK and aged-vehicle groups. Bacterial taxon showing a significant abundance of change (q-value < 0.001) was only shown with average fold-change value at the genus level. Red and blue indicate significantly increased bacterial taxa in young mice of 16s rRNA sequencing data and mice of 16s rRNA sequencing data, respectively. The Benjamini and Hochberg's FDR control method was used to correct for multiple comparisons. c, Analysis of differentially abundant microbial genus between AK-treated and untreated aged mice were analysed by LEfSe (Kruskal-Wallis test, *P* < 0.05, logarithmic LDA > 2.0). d, LEfSe analysis of KEGG enzymes in colon microbiota between AK-treated and untreated aged mice. No metabolic pathway is enriched between aged-AK and aged-vehicle groups. e, Survival rate and f, body weight change of AK-treated and untreated aged mice. g, Schematic summary shows TLR 2 and Wnt signalling pathways can be activated via the high abundance of Akkermansia in the aged-AK group compared to the aged-vehicle group. Lipopolysaccharides (LPS) derived from the membrane of Gram-negative bacteria are pro-inflammatory compounds. AMUC_1100 derived from *Akkermansia muciniphila* improves intestinal barrier function by increasing goblet cell density and stimulating Toll-like receptor 2 (TLR2). AMUC_1100 exerts a beneficial effect on mucus layer regeneration, inflammation, and insulin sensitivity. Wnt signalling is involved in the development and renewal of intestinal epithelium and hematopoietic stem cells. Arrow heads indicate stimulation and bar heads indicate inhibition.

**Additional file 2: Table S1.** Read statistics of 16s rRNA and metagenome sequencing.

**Additional file 3: Table S2.** Alpha and beta diversity analysis of the analysed groups in this study.

**Additional file 4: Table S3.** Taxa identified by LEfSe analysis in this study. "LogMaxMean" indicates the log of the highest-class average. "Enriched in" indicates the class with the highest mean if the taxa are discriminative. "LDA" indicates the logarithmic of the linear discriminant analysis (LDA) score. "P-value" indicates p-value of the pairwise Kruskal-Wallis test performed by LEfSe.

**Additional file 5: Table S4.** Effect of parabiotic pairing on blood parameters.

**Additional file 6: Table S5.** Relative abundance of phylotypes detected in ageing and rejuvenation samples.

**Additional file 7: Table S6.** Relative abundance of phylotypes detected in AK administration samples.

**Additional file 8: Table S7.** Frailty score used to develop a clinical frailty index in mice.

**Additional file 9: Table S8.** Sequences of PCR primers used in this study.

## Data Availability

Raw sequencing data of all 16S rRNA sequences and metagenomes have been deposited at the European Nucleotide Archive under the accession numbers PRJEB43096 and PRJEB43097, respectively.
