# Supplementary material for: Ageing and rejuvenation models reveal changes in key microbial communities associated with healthy ageing
Source: Microbiome. 2021 Dec 15;9:240. doi: 10.1186/s40168-021-01189-5 (PMC8672520; doi:10.1186/s40168-021-01189-5)
Supplement: Supplementary file 2 — Additional file 1: Note S1. Ageing-associated changes in microbial functional potential and metabolism. Note S2. Intestinal stem cell markers and de novo crypt formation in the colon of rejuvenated mice. Note S3. Administration of Akkermansia muciniphila improves canonical Wnt signalling and the senescence-related phenotype in the intestinal stem cell function and haematopoietic system. Fig. S1. Alpha-diversity and bacterial abundances at different ages. a–d, α-diversity based (a) Shannon, (b) observed OTU, (c) Faith's phylogenetic diversity, (d) evenness indices across the ageing process. Statistical testing showed a significant difference for Shannon diversity and observed species, while faith-PD richness was not significantly different in aged mice compared to young mice. Statistical analysis was performed using Kruskal-Wallis test (*, P < 0.05; **, P < 0.01). e–g, Heatmap for the bacteria abundances for the mice across ageing groups at the (e) order, (f) family, and (g) genus level. The taxonomic units with average abundance > 1% in each sampling time point are shown. Fig. S2. Beta-diversity at different ages. Principal coordinate analysis (PCoA) of β-diversity based weighted unifrac (left panel) and unweighted unifrac metric (right panel) across five ageing groups (P = 0.001; permutational multivariate analysis of variance, PERMANOVA). 100-week-old groups show significant differences with 4-week, 20-week, and 50-week-old groups (Table S2). Fig. S3. Taxonomic composition and difference during the ageing process determined by metagenomic sequencing. a, Taxonomic composition for colon microbiota composition determined by metagenomic sequencing at the genus level. The average relative abundance of the top 25 most abundant taxa in all samples is shown. b, A significant relative abundance change in response to ageing process. Bacterial taxon showing a significant abundance of change (q-value < 0.01) was only shown with average fold-change value at the genus level. Re [file 40168_2021_1189_MOESM2_ESM.pdf]

Note S1. Ageing-associated changes in microbial functional potential and metabolism.

First, pectin degradation-related genes in the pentose and glucuronate interconversion pathways (ko00040) were more abundant in the young group than in the aged group (**Fig. 1g** and **Fig. S4d**). Pectin can be fermented by the gut microbiota, producing SCFAs and changing the composition of the gut microbiota [1,2]. Furthermore, butyrate can be produced from lysine by a gut commensal [3,4], and the butyrate biosynthetic pathway is highly abundant in young mice. In the lysine degradation pathway (ko00310), D-alanine transaminase, 2-oxoglutarate dehydrogenase, and lysine 2,3-aminomutase showed higher levels (fold-change > 2.3, $q < 0.048$) in the young groups (**Fig. S4b**) than in the aged groups, resulting in a higher concentration of crotonyl-CoA. Butyrate can also be synthesised by inhibiting fatty acid elongation. β-ketoacyl ACP synthases are associated with the carbon chain elongation of fatty acids, and a low gene abundance and low expression of the *fabB* gene [5,6] or treatment with a specific inhibitor, such as cerulenin, cause the production of high levels of butyrate in bacteria [7]. The copy number of 3-oxoacyl-[acyl-carrier-protein] synthase I (FabB) in lysine degradation (ko00310) was the least significant (fold-change > 0.2, $q = 2.01 \times 10^{-14}$) in young mice (**Fig. S4e**), resulting in the possibility of increased accumulation of butyryl-CoA. The aforementioned crotonyl-CoA and butyryl-CoA can be converted to butyrate by key enzymes such as butyrate CoA transferase (butyryl-CoA:acetate CoA-transferase route) or butyrate kinase (classical pathway) [8]. Interestingly, the key enzyme of the butyrate-CoA transferase-mediated pathway was 7.2-fold more abundant in the young group than in the aged group (**Fig. S4g**), while phosphotransbutyrylase and butyrate-CoA transferase were less abundant (fold-change > 0.6, $q < 0.044$) in butanoate metabolism (ko00650), suggesting that this pathway may be the major butyrate biosynthesis pathway in the young group. These metagenomic data suggest that intestinal SCFA production decreases as fibre intake is lower in older subjects than in young subjects [9].

Moreover, the histidine metabolism (ko00340) was significantly abundant in the young mice, while the KEGG pathway of glutathione metabolism (ko00480) was abundant in the aged mice (**Fig. 1g**). These changes resulted in high glutamate accumulation in the young mice (**Fig. S4c** and **S4f**). Synthesised glutamate can be converted to GABA by glutamate decarboxylase (GAD), which is significantly abundant (fold-change = 1.6, $q = 0.006$) in the young mice. A previous report on metagenomic data from the Human Microbiome Project showed that a significant proportion of GAD genes is present in the human gut microbiota [10] and that GABA increases glucose tolerance and insulin sensitivity by inhibiting the inflammatory response of endotoxemia and protecting pancreatic β cells [11,12]. Consistently, our metagenomic analysis also showed that high levels of glutamate, which was significantly abundant in the young and rejuvenated groups, could be converted to GABA by GAD, suggesting that the microbial production of GABA may improve glucose tolerance and insulin sensitivity. These findings clearly indicate that a significant microbiome shift occurs, and the specific functional potential related to butyrate biosynthesis and GABA biosynthesis significantly changes in response to host ageing.

Note S2. Intestinal stem cell markers and de novo crypt formation in the colon of rejuvenated mice.

A recent study reported that ageing results in a decline in intestinal stem cell (ISC) function and impaired regenerative capacity of the intestinal epithelium [13]. We determined the expression of the ISC markers Lgr5 (CBC cell marker), Bmi1, and Hopx (+4 quiescent ISC marker) by qPCR analysis (**Fig. 2c**). Aged mice showed a significant decrease in colonic Lgr5 expression compared to young mice (two-tailed Student's *t*-test, $P < 0.05$). However, the expression of Bmi1 was significantly increased in aged mice from the co-housing experiment (two-tailed Student's *t*-test, $P < 0.05$, **Fig. 2c**). In the parabiosis experiment, the expression of both Lgr5 and Hopx was remarkably downregulated in the colon of isochronic aged pairs compared to the mice of isochronic young pairs, and heterochronic parabiosis led to a significant upregulation of these genes in aged mice (fold-change > 2, two-tailed Student's *t*-test, $P < 0.05$). We observed no significant changes in colonic ISC gene expression between young and aged mice in the serum injection model.

ISC function is altered due to reduced canonical Wnt signalling during ageing. To investigate the effects of rejuvenation on intestinal homeostasis, we evaluated the expression of the target genes of canonical Wnt signalling and that of the genes regulating ISC function in each colon sample from one ageing model and three experimental rejuvenation models. Co-housing of aged mice with young mice significantly increased the expression levels of *Ascl2*, *Cd44*, and *Wnt3* (two-tailed Student's *t*-test, $P < 0.05$) (**Fig. S5e**), whereas no significant changes (two-tailed Student's *t*-test, $P > 0.05$) were observed in the colonic gene expression between young and aged mice in the ageing and serum injection model (**Fig. S5d** and **S5g**). Interestingly, the expression levels of β-catenin, *Myc, Axin2, Olfm4*, and Cyclin D1 were significantly decreased in iso-aged mice compared to those in iso-young mice (two-tailed Student's *t*-test, $P < 0.05$) (**Fig. S5f**). In contrast, their aged counterparts from the hetero-aged group showed phenotypic restoration upon the mRNA expression of these genes compared to the aged mice from the iso-aged group. Additionally, we evaluated the extent of differences in cell proliferation in the intestinal crypts of young and aged mice using Ki67 IHC staining (**Fig. S5h** and **S5i**). CBC cells were frequently positive for the proliferation marker Ki67, indicating that the cells were actively cycling [14]. Ki67-positive cells per colonic crypt were present in lower numbers in the intestines of aged mice than in young mouse intestines; however, the colons of aged mice from heterochronic parabiosis restored Ki67-positive cells to a similar degree as seen in isochronic young mice (two-tailed Student's *t*-test, $P > 0.05$). These findings indicate that intestinal homeostasis might be altered upon ageing, and rejuvenating changes induced by parabiosis might promote de novo crypt formation in the colon of aged mice.

Note S3. Administration of *Akkermansia muciniphila* improves canonical Wnt signalling and the senescence-related phenotype in the intestinal stem cell function and haematopoietic system.

To evaluate whether AK treatment restores changes in canonical Wnt signalling and ISC function by ageing, the mRNA expression of Wnt target genes and ISC function-regulating genes were compared. The target genes exhibited a high level of expression in the colon samples obtained from the AK-treated group compared with those obtained from the untreated group (two-tailed Student's *t*-test, $P < 0.05$) (**Fig. S12a**). These results demonstrate that oral administration of AK improves impaired intestinal homeostasis due to ageing by accelerating the renewal and turnover of intestinal cells.

Since the ageing of haematopoietic stem cells (HSCs) is a key process in the decline of immune function with age or aging-associated diseases, we analysed the composition of lineage c-kit+Sca-1+ (LSK) cells using fluorescence-activated cell sorting in the bone marrow (BM). It has been reported that aged mice exhibit remarkable changes, such as functional deficits in reconstitution potential, expansion of CD34-Flk2-LSK cells (long-term haematopoietic stem cells; LT-HSCs), and lineage skewing with increased number of myeloid cells and decreased number of lymphoid cells [15] in their haematopoietic systems. Our data showed that the percentage of both short-term haematopoietic stem cells (ST-HSCs) and multipotential progenitors (MPPs) was comparable between the AK-treated and -untreated groups, while the proportion of LT-HSCs was significantly reduced (two-tailed Student's *t*-test, $P < 0.05$) by AK gavage (**Fig. S12b** and **S12d**). Interestingly, AK treatment reduced the proportion of neutrophils, which are representative myeloid cells (two-tailed Student's *t*-test, $P < 0.05$) (**Fig. S12c** and **S12e**). Regarding lymphoid cells, the percentage of B cells was significantly elevated by AK treatment, while the proportion of T cells was comparable between the AK-treated and -untreated groups. The BM niche plays a critical role in controlling the fate of HSCs by integrating intrinsic and extrinsic signals and can regulate both the self-renewal and multilineage differentiation of HSCs. It has been reported that intercellular adhesion molecule-1 (ICAM-1) maintains HSC quiescence and repopulation capacity in the BM niche, and ICAM-1 deficiency in the BM niche enhances the expansion of LT-HSCs and leads to an increase in the number of myeloid cells as well as a decrease in the number of lymphoid cells [16]. To identify a factor controlling the ageing phenotype of the haematopoietic system by AK, we performed gene expression analysis by isolating the BM niche, lineage-negative cells, and lineage-positive cells from the bone. Intriguingly, AK significantly elevated *Icam-1* expression only in the BM niche, while it was comparable in both lineage-negative and lineage-positive cells (**Fig. S12f**). These results suggest that AK ameliorates the senescence-related phenotype in the haematopoietic system by controlling ICAM-1 expression, particularly in the BM niche of mice.

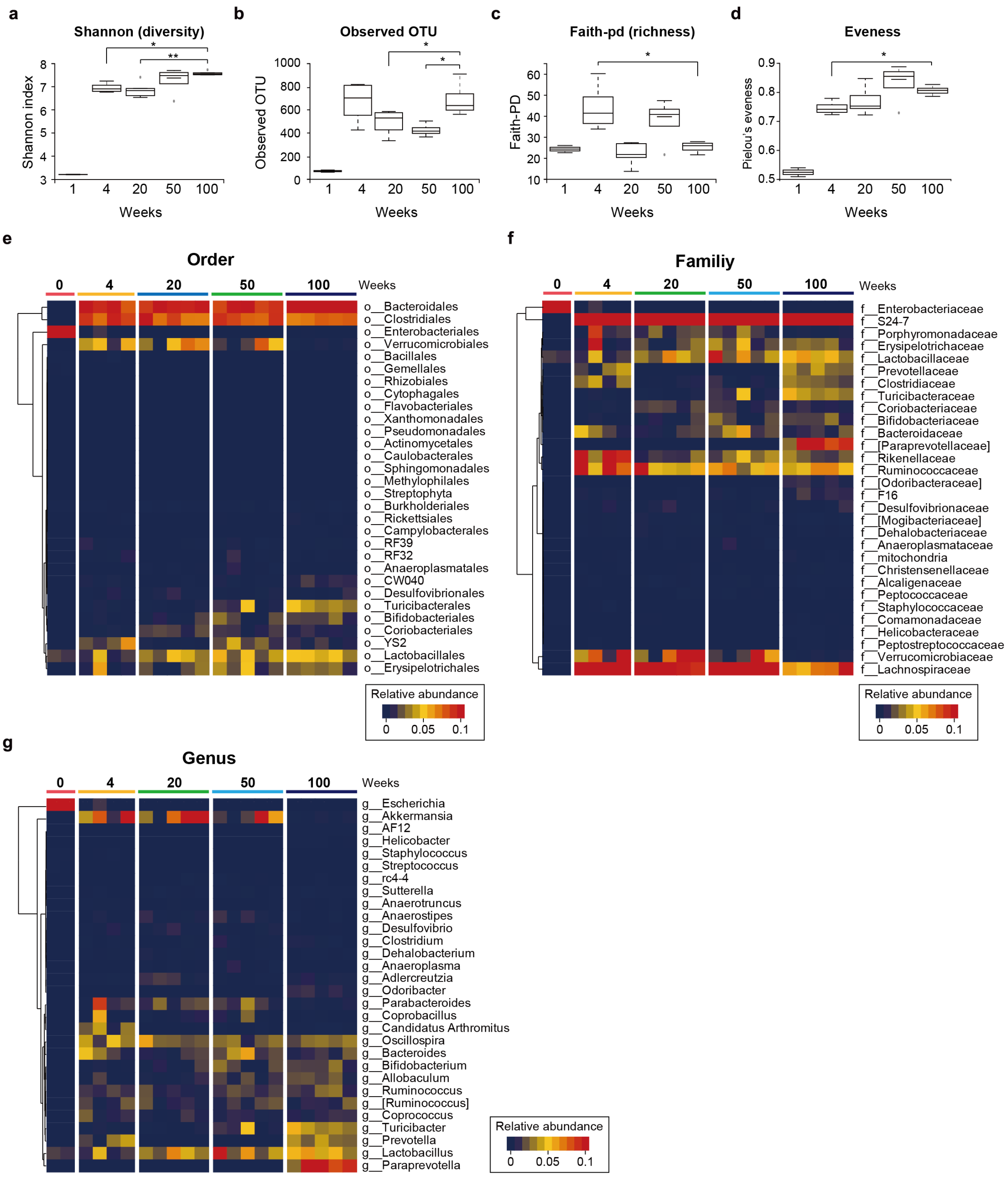

Fig. S1. Alpha-diversity and bacterial abundances at different ages. a–d, α-diversity based (**a**) Shannon, (**b**) observed OTU, (**c**) Faith's phylogenetic diversity, (**d**) evenness indices across the ageing process. Statistical testing showed a significant difference for Shannon diversity and observed species, while faith-PD richness was not significantly different in aged mice compared to young mice. Statistical analysis was performed using Kruskal-Wallis test (*, $P < 0.05$; **, $P < 0.01$). **e–g,** Heatmap for the bacteria abundances for the mice across ageing groups at the (**e**) order, (**f**) family, and (**g**) genus level. The taxonomic

units with average abundance > 1% in each sampling time point are shown.

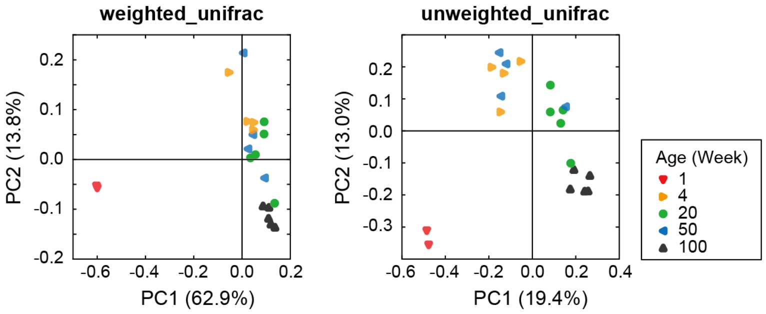

Fig. S2. Beta-diversity at different ages. Principal coordinate analysis (PCoA) of β-diversity based weighted unifrac (left panel) and unweighted unifrac metric (right panel) across five ageing groups ($P = 0.001$; permutational multivariate analysis of variance, PERMANOVA). 100-week-old groups show significant differences with 4-week, 20-week, and 50-week-old groups (**Table S2**).

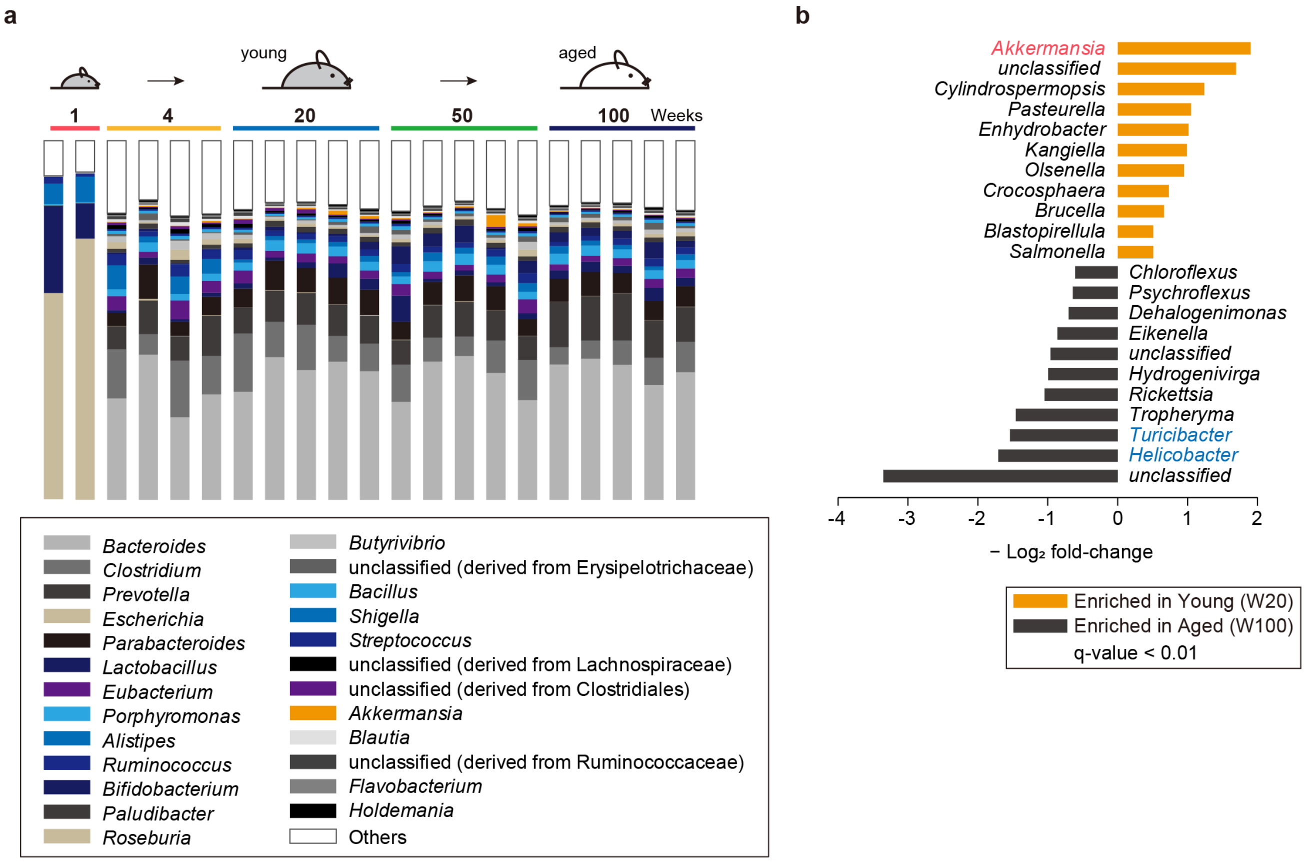

Fig. S3. Taxonomic composition and difference during the ageing process determined by metagenomic sequencing. a, Taxonomic composition for colon microbiota composition determined by metagenomic sequencing at the genus level. The average relative abundance of the top 25 most abundant taxa in all samples is shown. **b**, A significant relative abundance change in response to ageing process. Bacterial taxon showing a significant abundance of change (q-value < 0.01) was only shown with average fold-change value at the genus level. Red and blue indicate significantly increased bacterial taxa in young mice (W20) of 16s rRNA sequencing data and aged mice (W100) of 16s rRNA sequencing data, respectively. The Benjamini and Hochberg's FDR control method was used to correct for multiple comparisons.

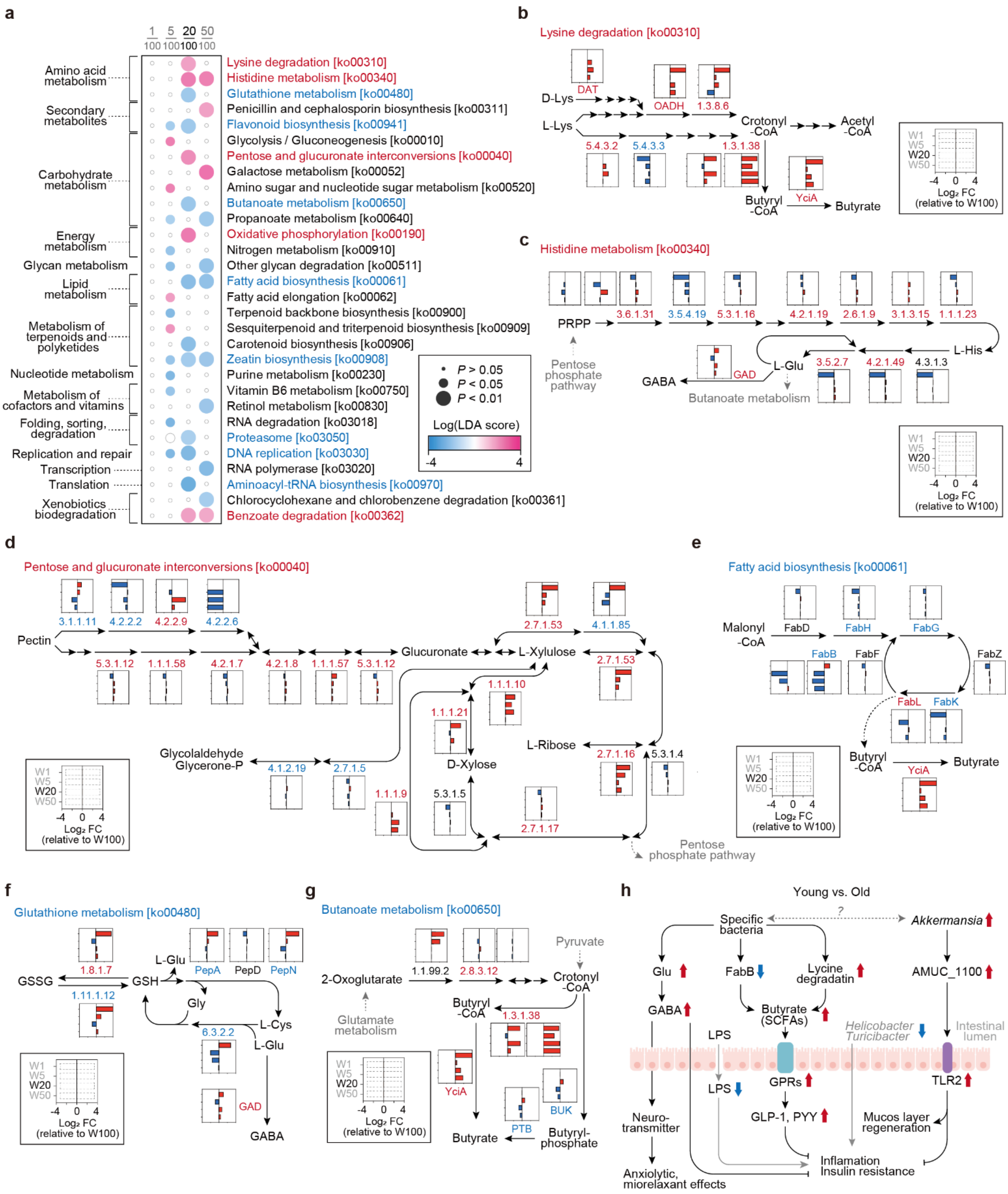

Fig. S4. Ageing-associated changes in microbial functional potential and metabolism. a, LEfSe analysis of metabolic pathways in colon samples from young group compared to aged group are shown. The four columns on the left show logarithmic linear discriminant analysis (LDA) score of KEGG metabolic pathway, comparing the 1-week, 4-week, 20-week, and 50-week-aged groups to 100-week-old group. Red and blue pathway indicate enriched metabolic

pathway in young mice and in aged mice, respectively. **b**–**g**, Enriched metabolic pathway presented in young mice compared to aged mice. Horizontal bar plot reflect the fold-change of each enzyme involved in (**b**) lysine degradation, (**c**) histidine metabolism, (**d**) pentose and glucuronate interconversions, (**e**) fatty acid biosynthesis, (**f**) glutathione metabolism, (**g**) and butanoate metabolism in 1-week, 4-week, 20-week, and 50-week-old groups compared to 100-week-old group. Red and blue KEGG reactions indicate high abundance in young mice and in aged mice, respectively. The following genes are represented by enzyme name and EC number: OADH, 2-oxoglutarate dehydrogenase (OADH) [EC:2.3.1.61]; DAT, D-alanine transaminase [EC:2.6.1.21]; glutaryl-CoA dehydrogenase [EC:1.3.8.6]; lysine 2,3-aminomutase [EC:5.4.3.2]; beta-lysine 5,6-aminomutase [EC:5.4.3.3]; trans-2-enoyl-CoA reductase [EC:1.3.1.38]; acyl-CoA thioesterase YciA [EC:3.1.2.-]; PRPP, phosphoribosyl pyrophosphate; phosphoribosyl-ATP pyrophosphohydrolase [EC:3.6.1.31]; phosphoribosyl-AMP cyclohydrolase [EC:3.5.4.19]; phosphoribosylformimino-5-aminoimidazole carboxamide ribotide isomerase [EC:5.3.1.16]; imidazoleglycerol-phosphate dehydratase [EC:4.2.1.19]; histidinol-phosphate aminotransferase [EC:2.6.1.9]; histidinol-phosphatase (PHP family) [EC:3.1.3.15]; histidinol dehydrogenase [EC:1.1.1.23]; histidine ammonia-lyase [EC:4.3.1.3]; urocanate hydratase [EC:4.2.1.49]; imidazolonepropionase [EC:3.5.2.7]; GAD, glutamate decarboxylase [EC:4.1.1.15]; pectinesterase [EC:3.1.1.11]; pectate lyase [EC:4.2.2.2]; pectate disaccharide-lyase [EC:4.2.2.9]; oligogalacturonide lyase [EC:4.2.2.6]; glucuronate isomerase [EC:5.3.1.12]; tagaturonate reductase [EC:1.1.1.58]; altronate hydrolase [EC:4.2.1.7]; mannonate dehydratase [EC:4.2.1.8]; fructuronate reductase [EC:1.1.1.57]; L-xylulokinase [EC:2.7.1.53]; 3-dehydro-L-gulonate-6-phosphate decarboxylase [EC:4.1.1.85]; L-xylulokinase [EC:2.7.1.53]; L-ribulokinase [EC:2.7.1.16]; L-arabinose isomerase [EC:5.3.1.4]; xylulokinase [EC:2.7.1.17]; L-xylulose reductase [EC:1.1.1.10]; aldehyde reductase [EC:1.1.1.21]; D-xylulose reductase [EC:1.1.1.9]; rhamnulokinase [EC:2.7.1.5]; rhamnulose-1-phosphate aldolase [EC:4.1.2.19]; FabD, [acyl-carrier-protein] S-malonyltransferase [EC:2.3.1.39]; FabH, 3-oxoacyl-[acyl-carrier-protein] synthase III [EC:2.3.1.180]; FabG, 3-oxoacyl-[acyl-carrier protein] reductase [EC:1.1.1.100]; FabZ, 3-hydroxyacyl-[acyl-carrier-protein] dehydratase [EC:4.2.1.59]; FabK, enoyl-[acyl-carrier protein] reductase II [EC:1.3.1.-]; FabL, enoyl-[acyl-carrier protein] reductase III [EC:1.3.1.-]; FabF, 3-oxoacyl-[acyl-carrier-protein] synthase II [EC:2.3.1.179]; FabB, 3-oxoacyl-[acyl-carrier-protein] synthase I [EC:2.3.1.41]; YciA, acyl-CoA thioesterase [EC:3.1.2.-]; GSR, glutathione reductase (NADPH) [EC:1.8.1.7]; phospholipid-hydroperoxide glutathione peroxidase [EC:1.11.1.12]; PepA, leucyl aminopeptidase [EC:3.4.11.1]; PepD; dipeptidase D [EC:3.4.13.-]; PepN; aminopeptidase N [EC:3.4.11.2]; glutamate--cysteine ligase [EC:6.3.2.2]; GAD, glutamate decarboxylase [EC:4.1.1.15]; 2-hydroxyglutarate dehydrogenase [EC:1.1.99.2]; glutaconate CoA-transferase [EC:2.8.3.12]; ptb; phosphate butyryltransferase [EC:2.3.1.19]; buk; butyrate kinase [EC:2.7.2.7]; trans-2-enoyl-CoA reductase [EC:1.3.1.38]; acyl-CoA thioesterase YciA [EC:3.1.2.-]. **h**, Schematic summary shows key metabolic differences between young and aged group based on relative gene abundance profiles. Host metabolism is influenced by γ-aminobutyric acid (GABA) neurotransmitter, affecting the brain (inducing satiety) GABA modulates inflammation. Fermentation of pectin by intestinal-specific bacteria produces butyrate. It affects host metabolism in several ways by acting on the G protein-coupled receptor (GPR) expressed by intestinal endocrine cells. Butyrate stimulate the release of glucagon-like peptide 1 (GLP-1) and peptide YY (PYY), affecting the pancreas (inducing insulin secretion) and brain (inducing satiety). Lipopolysaccharides (LPS) derived from the membrane of Gram-negative bacteria are pro-inflammatory compounds. AMUC_1100 derived from *Akkermansia muciniphila* improves intestinal barrier function by increasing goblet cell density and

stimulating Toll-like receptor 2 (TLR2). AMUC_1100 exerts the beneficial effect on mucus layer regeneration, inflammation, and insulin sensitivity. Arrow heads indicate stimulation and bar heads indicate inhibition.

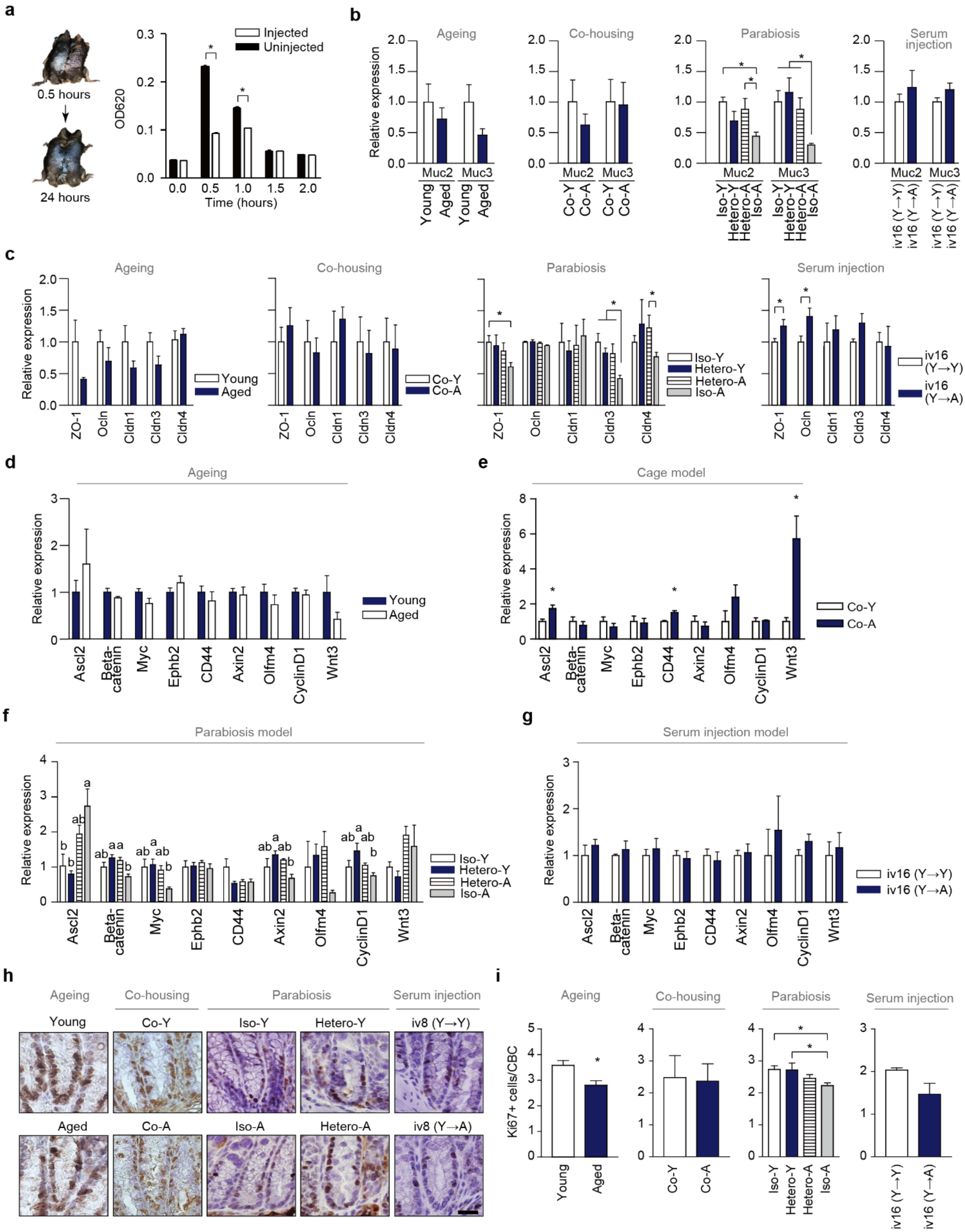

Fig. S5. Parabiosis experiments restores intestinal function, canonical Wnt signalling target genes, and genes regulating ISC function. a, Verification of blood sharing between the parabiotic pairs using Evans blue. Representative photographs of the mice were taken at

0.5 and 24 h after the injection of Evans blue into the tail vein of one parabiont in a pair a week after surgery. The serum concentration of Evans blue in both mice in each pair was measured at 620 nm by spectrophotometry at 0, 0.5, 1, 24, and 48 h after injection. **b**, Gene expression profile of mucin in the colon. **c**, Gene expression profile of barrier-forming tight junction proteins in the colon. **d–g**, Quantitative real-time PCR analyses for expression of canonical Wnt signalling target genes and genes regulating ISC function in ageing, cage model, serum injection model and parabiosis model. Data are means ± SEMs (*, $P < 0.05$, two-tailed Student's t-test). ^{ab} means not sharing a common letter are significantly different at $P < 0.05$. **h**, Representative Ki67-stained pictures. Scale bar, 25 μm. **i**, quantification of Ki-67 positive cells per crypt base columnar cell in the colon. Data are means ± SEM. (*, $P < 0.05$, two-tailed Student's t-test).

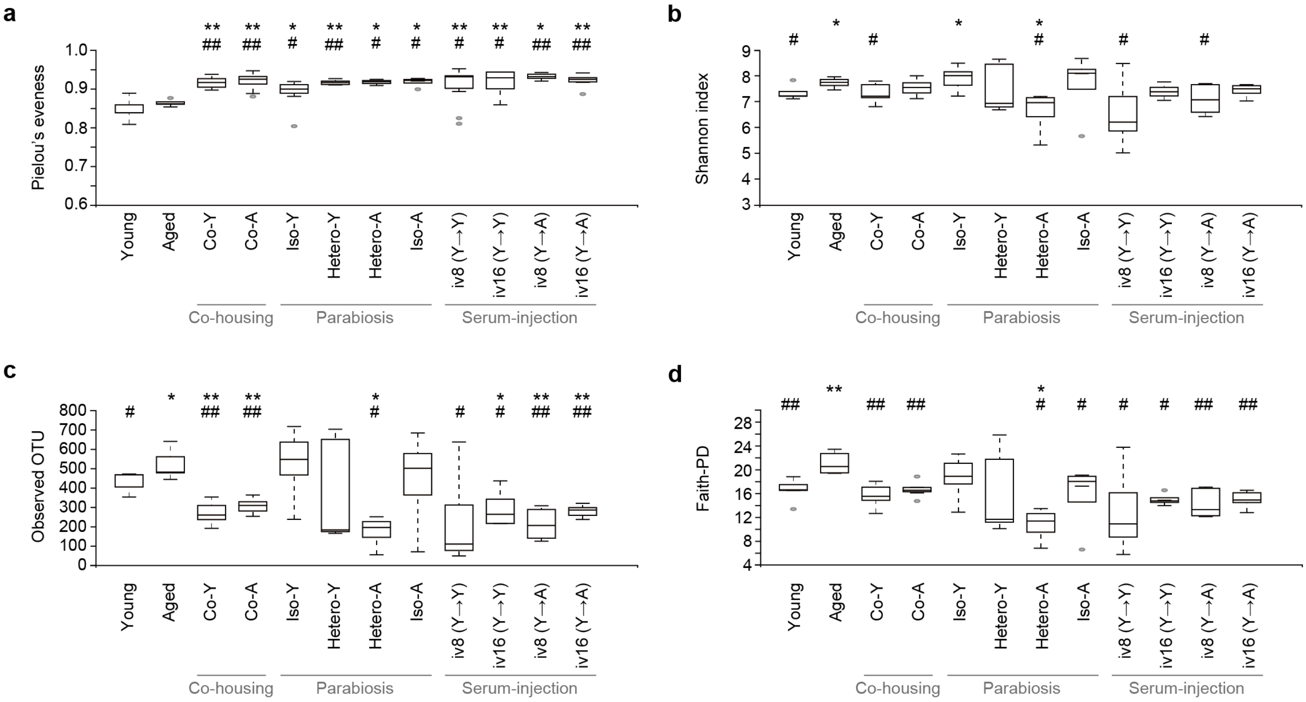

Fig. S6. Dynamics of α-diversity indices including (a) Shannon diversity, (b) observed OTU, (c) Faith's phylogenetic diversity, (d) Pielou's evenness indices among samples of the co-housing, parabiosis, and serum injection groups. Statistical analysis was performed using Kruskal-Wallis test, young (week 20) versus rejuvenated mice group (*, $P < 0.05$; **, $P < 0.01$); aged (W100) versus rejuvenated mice group (#, $P < 0.05$; ##, $P < 0.01$). Abbreviations: Co-Y, young mice from co-housing experiments; Co-A, aged mice from co-housing experiments; Hetero-Y, young mice from heterochronic pairs; Hetero-A, aged mice from heterochronic pairs; Iso-Y, young mice from isochronic young pairs; Iso-A, aged mice from isochronic aged pairs; iv 8 (Y→Y), young mice treated with serum isolated from young mice by intravenously into the tail vein 8 times; iv 16 (Y→Y), young mice treated with serum isolated from young mice by intravenously into the tail vein 16 times; iv 8 (Y→A), aged mice treated with serum isolated from young mice by intravenously into the tail vein 8 times; iv 16 (Y→A), aged mice treated with serum isolated from young mice by intravenously into the tail vein 16 times.

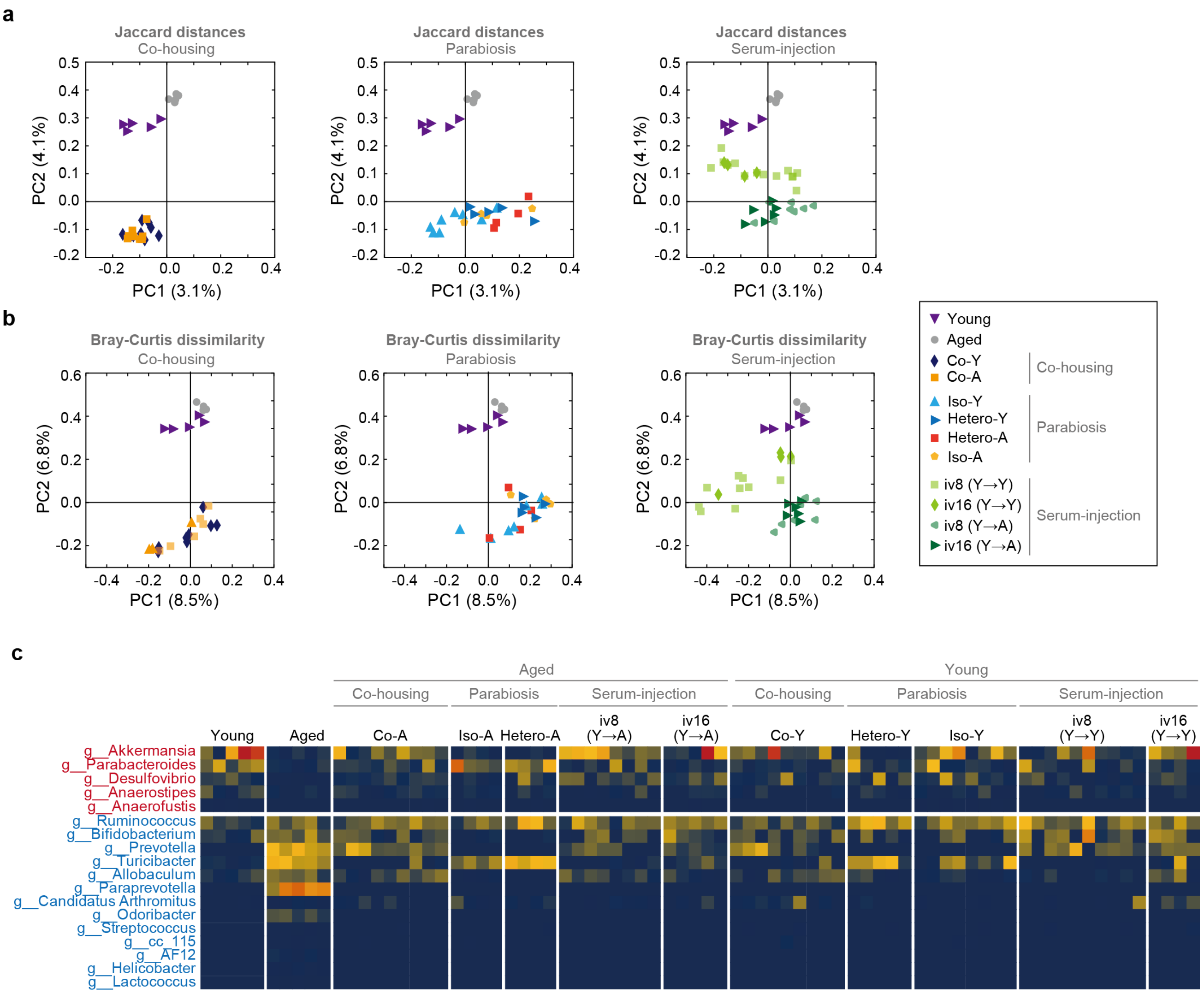

Fig. S7. Gut microbiome alteration in several rejuvenation models. a–b, Principal coordinate analysis (PCoA) of β-diversity based (**a**) Jaccard distances, and (**b**) Bray-Curtis dissimilarity metric among samples of the co-housing (left), parabiosis (middle) and serum injection (right) groups of mice analyzed (PERMANOVA, $P = 0.001$). Each dot represents an individual mouse. **c,** Heatmap presenting the relative abundance (%) of the key prevalent bacterial taxa found in young and aged-mice. The red and blue genus indicates enriched bacterial taxa in young mice (W20) and aged mice (W100), respectively.

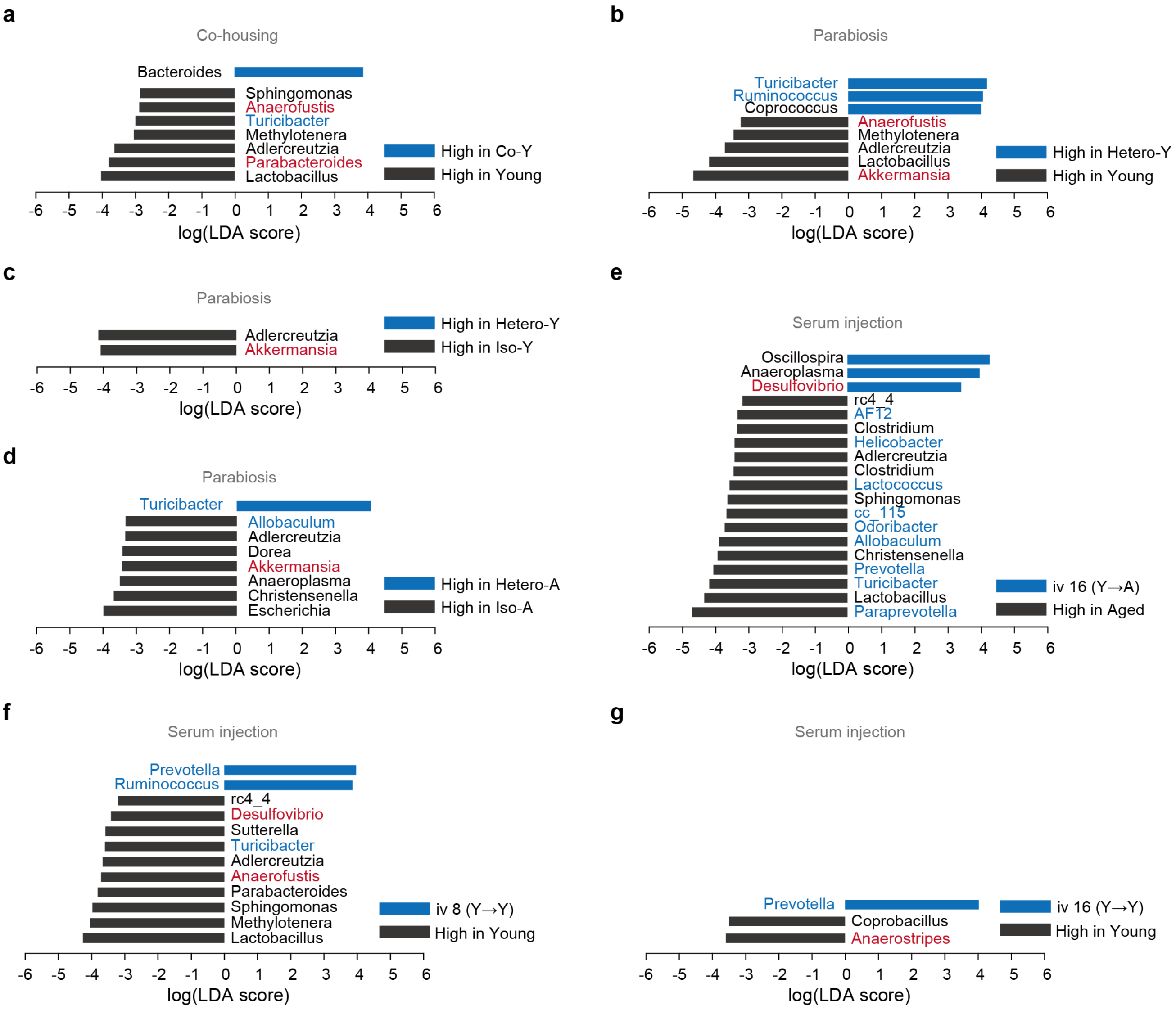

Fig. S8. LEfSe analysis showing microbial genus that was significantly different in abundance between (a) young mice (W20) and Co-Y, (b) young mice and Hetero-Y, (c) Hetero-Y mice and Iso-Y, (d) Hetero-A and Iso-A, (e) aged mice and iv 16 (Y→A), (f) young mice and iv 8 (Y→Y), and (g) young mice and iv 16 (Y→Y). Abbreviations: Co-Y, young mice from co-housing experiments; Hetero-Y, young mice from heterochronic pairs; Hetero-A, aged mice from heterochronic pairs; Iso-Y, young mice from isochronic young pairs; Iso-A, aged mice from isochronic aged pairs; iv 8 (Y→Y), young mice treated with serum isolated from young mice by intravenously into the tail vein 8 times; iv 16 (Y→Y), young mice treated with serum isolated from young mice by intravenously into the tail vein 16 times; iv 16 (Y→A), aged mice treated with serum isolated from young mice by intravenously into the tail vein 16 times. Abbreviations: Co-Y, young mice from co-housing experiments; Co-A, aged mice from co-housing experiments; Hetero-Y, young mice from heterochronic pairs; Hetero-A, aged mice from heterochronic pairs; Iso-Y, young mice from isochronic young pairs; Iso-A, aged mice from isochronic aged pairs; iv 8 (Y→Y), young mice treated with serum isolated from young mice by intravenously into the tail vein 8 times; iv 16 (Y→Y), young mice treated with serum isolated from young mice by intravenously into the tail vein 16 times; iv 8 (Y→A), aged mice treated with serum isolated from young mice

by intravenously into the tail vein 8 times; iv 16 (Y→A), aged mice treated with serum isolated from young mice by intravenously into the tail vein 16 times.

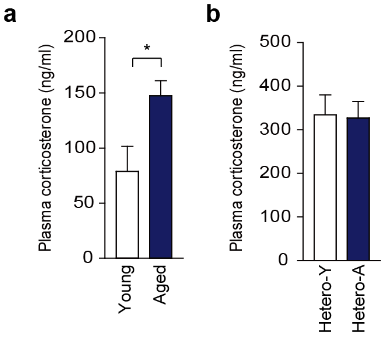

Fig. S9. Parabiosis experiments induce corticosterone levels in plasma. Plasma corticosterone levels in (**a**) naïve mice (young and aged mice) and (**b**) heterochronic parabiotic paired mice. All data are means ± SEMs (Mann-Whitney U test; *, $P < 0.05$).

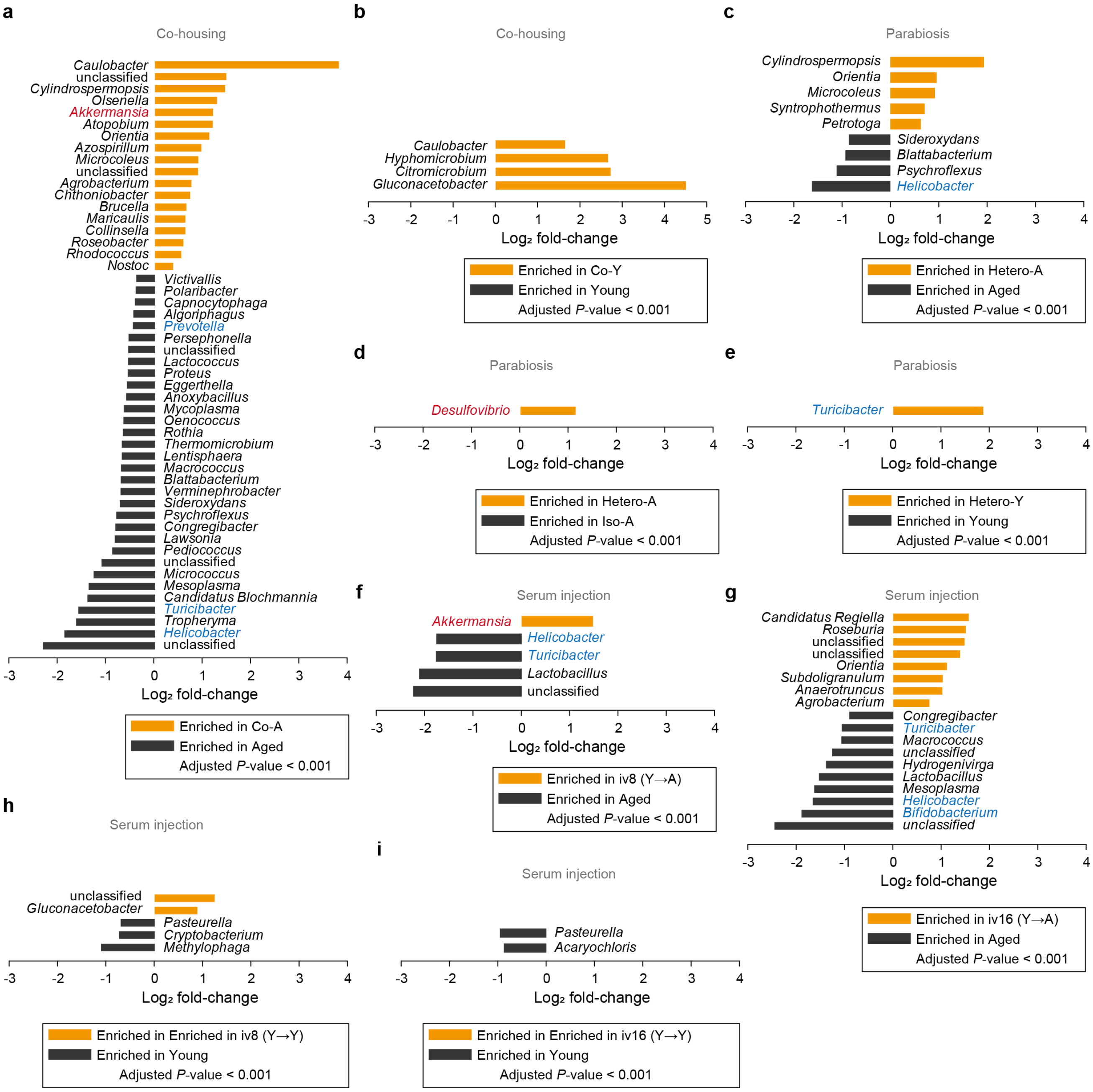

Fig. S10. Taxonomic composition and difference during rejuvenation process determined by metagenomic sequencing. Several microbial genera were significantly different in abundance between (**a**) Co-A and aged, (**b**) Co-Y and young, (**c**) Hetero-A and aged, (**d**) Hetero-A and Iso-A, (**e**) Hetero-Y and Young, (**f**) iv 8 (Y→A) and aged, (**g**) iv 16 (Y→A) and aged, (**h**) iv 8 (Y→Y) and young, and (**i**) iv 16 (Y→Y) and young. Bacterial taxon showing a significant abundance of change (q-value < 0.001) was only shown with average fold-change value at the genus level. Red and blue indicate significantly increased bacterial taxa in young mice of 16s rRNA sequencing data and mice of 16s rRNA sequencing data, respectively. The Benjamini and Hochberg's FDR control method was used to correct for multiple comparisons. Abbreviations: Co-Y, young mice from co-housing experiments; Co-A, aged mice from co-housing experiments; Hetero-Y, young mice from heterochronic pairs; Hetero-A, aged mice from heterochronic pairs; Iso-Y, young mice from isochronic young pairs; Iso-A, aged mice from isochronic aged pairs; iv 8 (Y→Y), young mice treated

with serum isolated from young mice by intravenously into the tail vein 8 times; iv 16 (Y→Y), young mice treated with serum isolated from young mice by intravenously into the tail vein 16 times; iv 8 (Y→A), aged mice treated with serum isolated from young mice by intravenously into the tail vein 8 times; iv 16 (Y→A), aged mice treated with serum isolated from young mice by intravenously into the tail vein 16 times.

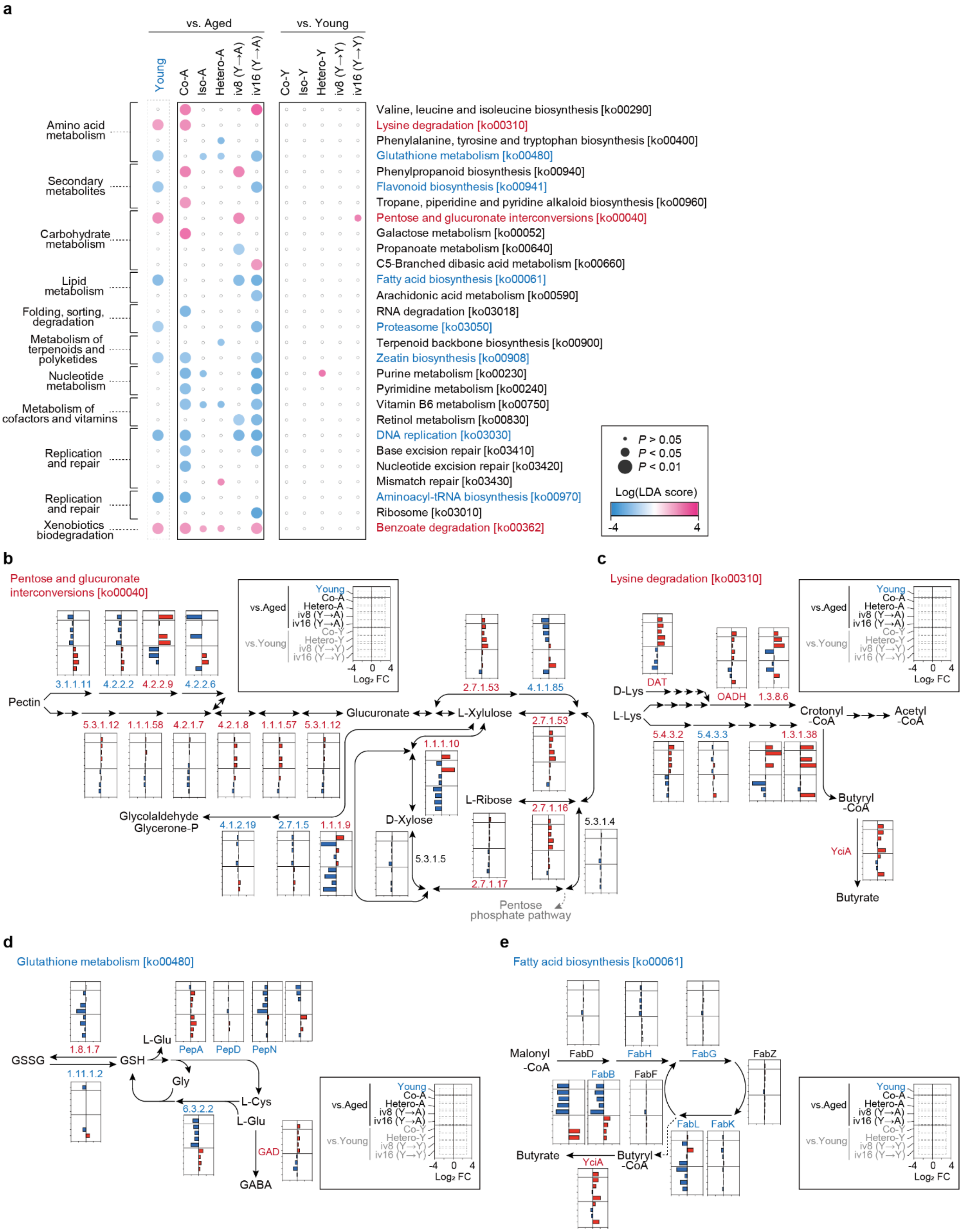

Fig. S11. Rejuvenation-associated changes in microbial functional potential and metabolism. a, LEfSe analysis of metabolic pathways in colon microbiota during each rejuvenation procedure. The two columns on the left indicate logarithmic LDA scores

categorized by KEGG pathways, comparing young group, Co-A, Iso-A, Hetero-A, iv 8 (Y→A), and iv 16 (Y→A) to the aged group. The last column on the right shows logarithmic LDA scores categorized by KEGG pathways, comparing Co-Y, Iso-Y, Hetero-Y, iv 8 (Y→Y), and iv 16 (Y→Y) to the young group. The red and blue names of pathways indicate enriched metabolic pathways in young mice and in aged mice, respectively. Only KEGG pathways differ significantly during each rejuvenation procedure. **b–e,** Shown horizontal bar plots indicate the fold-change of each enzyme involved in (**b**) pentose and glucuronate interconversions, (**c**) lysine degradation, (**d**) glutathione metabolism, and (**e**) fatty acid biosynthesis in young, Co-A, Hetero-A, iv8 (Y→A), iv16 (Y→A), Co-Y, Hetero-Y, iv8 (Y→Y), and iv16 (Y→Y). Red and blue KEGG reactions indicate high abundance in young mice and in aged mice, respectively. Abbreviations: Co-Y, young mice from co-housing experiments; Hetero-Y, young mice from heterochronic pairs; Hetero-A, aged mice from heterochronic pairs; Iso-Y, young mice from isochronic young pairs; Iso-A, aged mice from isochronic aged pairs; iv 8 (Y→Y), young mice treated with serum isolated from young mice by intravenously into the tail vein 8 times; iv 16 (Y→Y), young mice treated with serum isolated from young mice by intravenously into the tail vein 16 times; iv 16 (Y→A), aged mice treated with serum isolated from young mice by intravenously into the tail vein 16 times. The following genes are represented by enzyme name and EC number: OADH, 2-oxoglutarate dehydrogenase (OADH) [EC:2.3.1.61]; DAT, D-alanine transaminase [EC:2.6.1.21]; glutaryl-CoA dehydrogenase [EC:1.3.8.6]; lysine 2,3-aminomutase [EC:5.4.3.2]; beta-lysine 5,6-aminomutase [EC:5.4.3.3]; trans-2-enoyl-CoA reductase [EC:1.3.1.38]; acyl-CoA thioesterase YciA [EC:3.1.2.-]; GAD, glutamate decarboxylase [EC:4.1.1.15]; pectinesterase [EC:3.1.1.11]; pectate lyase [EC:4.2.2.2]; pectate disaccharide-lyase [EC:4.2.2.9]; oligogalacturonide lyase [EC:4.2.2.6]; glucuronate isomerase [EC:5.3.1.12]; tagaturonate reductase [EC:1.1.1.58]; altronate hydrolase [EC:4.2.1.7]; mannonate dehydratase [EC:4.2.1.8]; fructuronate reductase [EC:1.1.1.57]; L-xylulokinase [EC:2.7.1.53]; 3-dehydro-L-gulonate-6-phosphate decarboxylase [EC:4.1.1.85]; L-xylulokinase [EC:2.7.1.53]; L-ribulokinase [EC:2.7.1.16]; L-arabinose isomerase [EC:5.3.1.4]; xylulokinase [EC:2.7.1.17]; L-xylulose reductase [EC:1.1.1.10]; aldehyde reductase [EC:1.1.1.21]; D-xylulose reductase [EC:1.1.1.9]; rhamnulokinase [EC:2.7.1.5]; rhamnulose-1-phosphate aldolase [EC:4.1.2.19]; FabD, [acyl-carrier-protein] S-malonyltransferase [EC:2.3.1.39]; FabH, 3-oxoacyl-[acyl-carrier-protein] synthase III [EC:2.3.1.180]; FabG, 3-oxoacyl-[acyl-carrier protein] reductase [EC:1.1.1.100]; FabZ, 3-hydroxyacyl-[acyl-carrier-protein] dehydratase [EC:4.2.1.59]; FabK, enoyl-[acyl-carrier protein] reductase II [EC:1.3.1.-]; FabL, enoyl-[acyl-carrier protein] reductase III [EC:1.3.1.-]; FabF, 3-oxoacyl-[acyl-carrier-protein] synthase II [EC:2.3.1.179]; FabB, 3-oxoacyl-[acyl-carrier-protein] synthase I [EC:2.3.1.41]; YciA, acyl-CoA thioesterase [EC:3.1.2.-]; GSR, glutathione reductase (NADPH) [EC:1.8.1.7]; phospholipid-hydroperoxide glutathione peroxidase [EC:1.11.1.12]; PepA, leucyl aminopeptidase [EC:3.4.11.1]; PepD; dipeptidase D [EC:3.4.13.-]; PepN; aminopeptidase N [EC:3.4.11.2]; glutamate--cysteine ligase [EC:6.3.2.2]; GAD, glutamate decarboxylase [EC:4.1.1.15]; trans-2-enoyl-CoA reductase [EC:1.3.1.38]; acyl-CoA thioesterase YciA [EC:3.1.2.-].

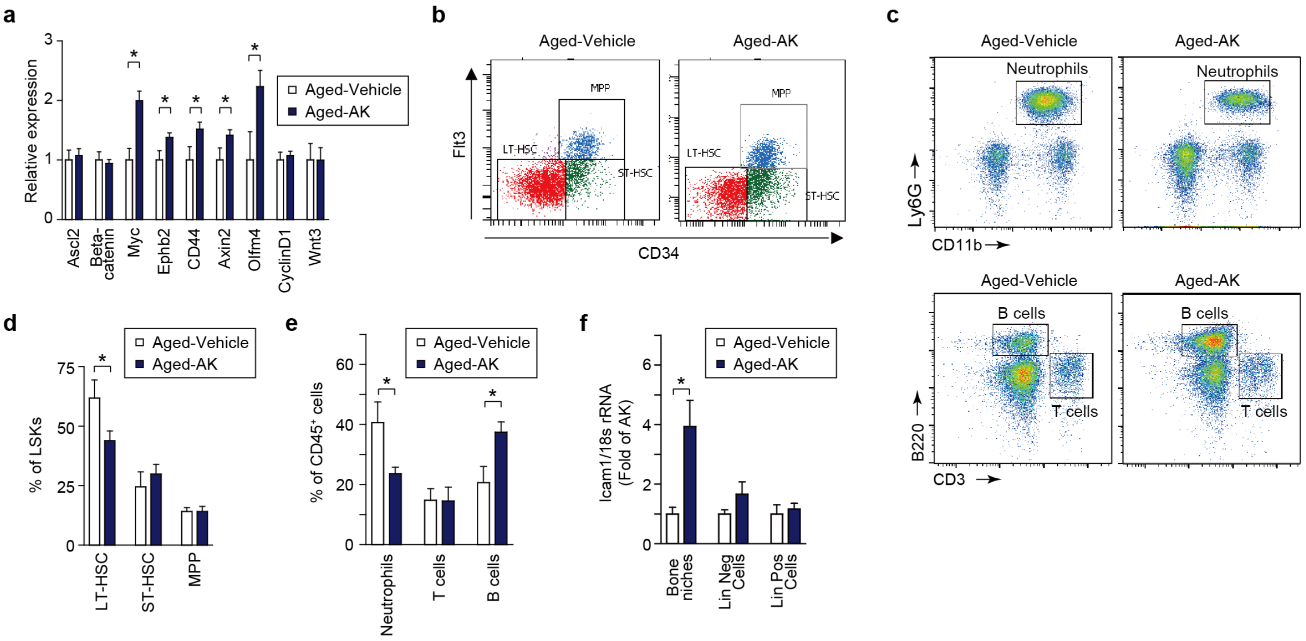

Fig. S12. Administration of AK restores the expression of the canonical Wnt signalling target genes and ameliorates the senescence-related phenotype in haematopoietic system. **a,** Quantitative reverse transcription PCR (qRT-PCR) analyses for the expression of canonical Wnt signalling target genes and genes regulating ISC function. Data are presented as means ± SEMs (*, $P < 0.05$, two-tailed Student's *t*-test). **b,** A representative fluorescence-activated cell sorting plot showing the frequencies of LT-HSCs, ST-HSCs, and MPPs among LSKs in the bone marrow of AK-treated and untreated aged mice. **c,** Percentages of LT-HSCs, ST-HSCs, and MPPs among LSKs. Data are presented means ± SEMs (*, $P < 0.05$, two-tailed Student's *t*-test). **d**, Representative images and **e,** frequencies of neutrophils, T cells, and B cells in the blood of AK-treated and untreated aged mice. Data are presented as means ± SEMs (*, $P < 0.05$, two-tailed Student's *t*-test). **f,** qRT-PCR analysis of the *Icam1* mRNA purified from bone niches and lineage-negative and -positive cells of the aged mice treated with AK. Data are presented as means ± SEMs (*, $P < 0.05$, two-tailed Student's *t*-test).

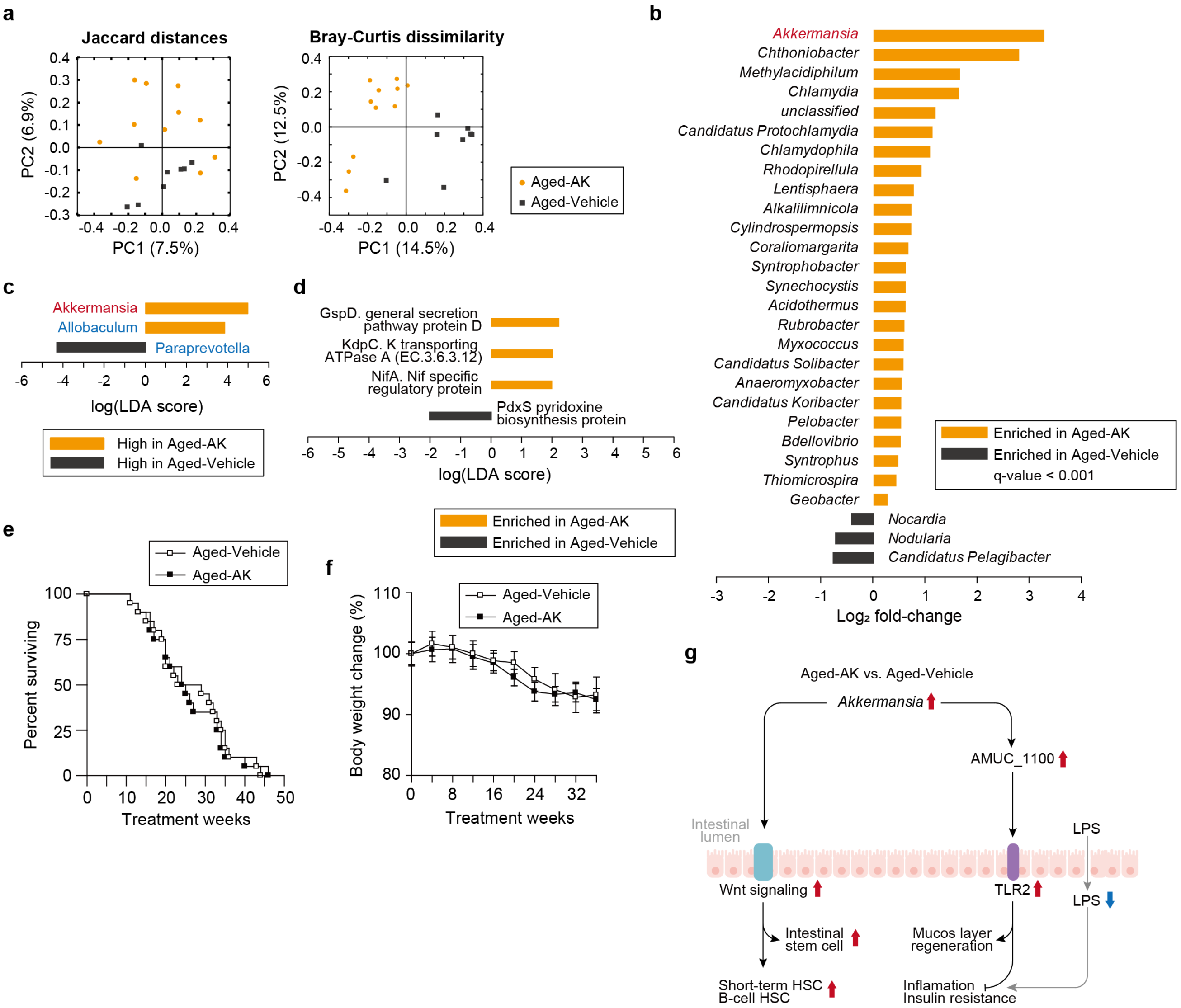

Fig. S13. Effect of AK treatment in aged mice. a, Principal coordinate analysis (PCoA) of β-diversity based Jaccard distances and Bray-Curtis dissimilarity metric between AK-treated and untreated aged mice ($P = 0.001$; permutational multivariate analysis of variance, PERMANOVA). The gut microbiome of AK-treated aged mice shows significant differences with control (**Table S2**). **b,** Taxonomic composition and difference determined by metagenomic sequencing. Microbial genera were significantly different in abundance between aged-AK and aged-vehicle groups. Bacterial taxon showing a significant abundance of change (q-value < 0.001) was only shown with average fold-change value at the genus level. Red and blue indicate significantly increased bacterial taxa in young mice of 16s rRNA sequencing data and mice of 16s rRNA sequencing data, respectively. The Benjamini and Hochberg's FDR control method was used to correct for multiple comparisons. **c,** Analysis of differentially abundant microbial genus between AK-treated and untreated aged mice were analyzed by LEfSe (Kruskal-Wallis test, $P < 0.05$, logarithmic LDA > 2.0). **d,** LEfSe analysis of KEGG enzymes in colon microbiota between AK-treated and untreated aged mice. No metabolic pathway is enriched between aged-AK and aged-vehicle groups. **e,** Survival rate and **f,** body

weight change of AK-treated and untreated aged mice. **g,** Schematic summary shows TLR 2 and Wnt signalling pathways can be activated via the high abundance of *Akkermansia* in the aged-AK group compared to the aged-vehicle group. Lipopolysaccharides (LPS) derived from the membrane of Gram-negative bacteria are pro-inflammatory compounds. AMUC_1100 derived from *Akkermansia muciniphila* improves intestinal barrier function by increasing goblet cell density and stimulating Toll-like receptor 2 (TLR2). AMUC_1100 exerts a beneficial effect on mucus layer regeneration, inflammation, and insulin sensitivity. Wnt signalling is involved in the development and renewal of intestinal epithelium and hematopoietic stem cells. Arrow heads indicate stimulation and bar heads indicate inhibition.

References

1. Duncan SH, Holtrop G, Lobley GE, Calder AG, Stewart CS, Flint HJ. Contribution of acetate to butyrate formation by human faecal bacteria. Brit J Nutr. 2004;91:915–23.
2. Bang S-J, Kim G, Lim MY, Song E-J, Jung D-H, Kum J-S, et al. The influence of in vitro pectin fermentation on the human fecal microbiome. Amb Express. 2018;8:98.
3. Bui TPN, Ritari J, Boeren S, Waard P de, Plugge CM, Vos WM de. Production of butyrate from lysine and the Amadori product fructoselysine by a human gut commensal. Nat Commun. 2015;6:10062.
4. Vital M, Howe AC, Tiedje JM. Revealing the Bacterial Butyrate Synthesis Pathways by Analyzing (Meta)genomic Data. Mbio. 2014;5:e00889-14.
5. Jawed K, Mattam AJ, Fatma Z, Wajid S, Abdin MZ, Yazdani SS. Engineered Production of Short Chain Fatty Acid in Escherichia coli Using Fatty Acid Synthesis Pathway. Plos One. 2016;11:e0160035.
6. Gong Y, Miao X. Short Chain Fatty Acid Biosynthesis in Microalgae Synechococcus sp. PCC 7942. Mar Drugs. 2019;17:255.
7. Torella JP, Ford TJ, Kim SN, Chen AM, Way JC, Silver PA. Tailored fatty acid synthesis via dynamic control of fatty acid elongation. Proc National Acad Sci. 2013;110:11290–5.
8. Louis P, Hold GL, Flint HJ. The gut microbiota, bacterial metabolites and colorectal cancer. Nat Rev Microbiol. 2014;12:661–72.
9. Tiihonen K, Ouwehand AC, Rautonen N. Human intestinal microbiota and healthy ageing. Ageing Res Rev. 2010;9:107–16.
10. Pokusaeva K, Johnson C, Luk B, Uribe G, Fu Y, Oezguen N, et al. GABA-producing Bifidobacterium dentium modulates visceral sensitivity in the intestine. Neurogastroenterol Motil. 2017;29:e12904.
11. Purwana I, Zheng J, Li X, Deurloo M, Son DO, Zhang Z, et al. GABA Promotes Human -Cell Proliferation and Modulates Glucose Homeostasis. Diabetes. 2014;63:4197–205.
12. Tian J, Dang HN, Yong J, Chui W-S, Dizon MPG, Yaw CKY, et al. Oral Treatment with γ-Aminobutyric Acid Improves Glucose Tolerance and Insulin Sensitivity by Inhibiting Inflammation in High Fat Diet-Fed Mice. Plos One. 2011;6:e25338.
13. Nalapareddy K, Nattamai KJ, Kumar RS, Karns R, Wikenheiser-Brokamp KA, Sampson LL, et al. Canonical Wnt Signaling Ameliorates Aging of Intestinal Stem Cells. Cell Reports. 2017;18:2608–21.
14. Basak O, Born M, Korving J, Beumer J, Elst S, Es JH, et al. Mapping early fate determination in Lgr5+ crypt stem cells using a novel Ki67-RFP allele. Embo J. 2014;33:2057–68.
15. Morrison SJ, Wandycz AM, Akashi K, Globerson A, Weissman IL. The aging of hematopoietic stem cells. Nat Med. 1996;2:1011–6.
16. Liu Y, Zhang S, Chen Y, Shi K, Zou B, Liu J, et al. ICAM-1 Deficiency in the Bone Marrow Niche Impairs Quiescence and Repopulation of Hematopoietic Stem Cells. Stem Cell Rep. 2018;11:258–73.
