# Supplementary material for: Ageing and rejuvenation models reveal changes in key microbial communities associated with healthy ageing
Source: Microbiome. 2021 Dec 15;9:240. doi: 10.1186/s40168-021-01189-5 (PMC8672520; doi:10.1186/s40168-021-01189-5)
Supplement: Supplementary file 3 — Additional file 2: Table S1. Read statistics of 16s rRNA and metagenome sequencing. [file 40168_2021_1189_MOESM3_ESM.pdf]

Table S1. Read statistics of 16s rRNA and metagenome sequencing.

Library	Sample name	Sample	Raw data	QIIME2
			Reads	Sequence variants
**16s rRNA library**	A16-16s	Aging (Week1)	759,054	192,011
	A17-16s		1,150,359	443,170
	A6-16s	Aging (Week4)	1,265,379	347,357
	A7-16s		656,935	113,601
	A8-16s		1,261,683	355,781
	A9-16s		790,104	214,215
	A1-16s	Aging (Week20)	339,830	80,670
	A2-16s		552,334	132,833
	A3-16s		1,234,048	266,863
	A4-16s		935,997	225,855
	A5-16s		824,221	210,852
	A11-16s	Aging (Week50)	400,141	85,757
	A12-16s		249,593	54,339
	A13-16s		258,439	21,617
	A14-16s		366,322	104,475
	A15-16s		183,029	45,305
	A24-16s	Aging (Week100)	453,212	115,669
	A25-16s		413,167	101,028
	A26-16s		369,154	83,746
	A27-16s		1,014,126	227,604
	A28-16s		1,049,964	250,802
	C1-16s	Co-housing Co-Young	136,575	26,169
	C2-16s		144,087	26,419
	C3-16s		161,722	35,917
	C4-16s		146,017	25,060
	C5-16s		136,247	35,962
	C6-16s		234,192	29,918
	C7-16s		151,688	35,025
	C8-16s		117,396	25,623
	C9-16s		61,853	15,597
	C10-16s	Co-housing Co-Aged	141,078	29,533
	C20-16s		124,279	28,113
	C30-16s		155,716	31,855

C40-16s		125,790	29,724
C50-16s		175,343	32,948
C60-16s		178,033	33,371
C70-16s		109,923	28,203
C80-16s		149,736	33,780
C90-16s		161,162	36,264
OY-Y1-16s	Parabiosis Hetero-Young	708,001	56,441
OY-Y2-16s		803,928	60,012
OY-Y3-16s		647,779	54,537
OY-Y4-16s		384,013	89,848
OY-Y5-16s		572,869	127,315
OY-O1-16s	Parabiosis Hetero-Aged	241,320	11,187
OY-O2-16s		848,270	68,856
OY-O3-16s		595,260	73,032
OY-O4-16s		936,483	109,548
YY1-16s	Parabiosis Iso-Young	244,059	42,432
YY2-16s		936,833	77,717
YY3-16s		357,921	77,464
YY4-16s		321,752	87,203
YY5-16s		423,085	131,791
YY6-16s		1,002,458	231,947
YY7-16s		843,748	251,719
YY8-16s		472,525	117,938
OO1-16s	Parabiosis Iso-Aged	346,107	24,283
OO2-16s		257,536	60,415
OO3-16s		614,589	117,453
OO4-16s		804,066	124,271
Serum-8iv-8-01	Serum-injection Young to Aged (iv8)	52,511	10,782
Serum-8iv-8-02		52,548	12,154
Serum-8iv-8-03		60,348	10,731
Serum-8iv-8-12		82,408	13,931
Serum-16iv-8-04		168,148	26,764
Serum-16iv-8-05		113,574	21,447
Serum-16iv-8-14		129,719	26,435
Serum-16iv-8-15		79,199	20,087
Serum-16iv-8-16		124,788	29,158
Serum-16iv-16-04		105,379	20,383

	Serum-16iv-16-05	Serum-injection Young to Aged (iv16)	130,625	23,848
	Serum-16iv-16-14		138,906	32,002
	Serum-16iv-16-15		103,904	24,414
	Serum-16iv-16-16		133,815	30,396
	YtoY8-11-16s	Serum-injection Young to Young (iv8)	35,055	3,844
	YtoY8-1-16s		34,296	4,650
	YtoY8-2-16s		149,244	24,322
	YtoY8-3-16s		35,811	5,061
	YtoY8-4-16s		37,931	3,728
	YtoY8-5-16s		67,985	2,877
	YtoY8-6-16s		1,203,921	180,696
	YtoY8-7-16s		67,991	14,843
	YtoY8-8-16s		266,256	41,533
	YtoY8-9-16s		32,111	4,712
	YtoY16-6-16s	Serum-injection Young to Young (iv16)	75,277	14,950
	YtoY16-7-16s		93,962	14,791
	YtoY16-8-16s		67,436	10,956
	YtoY16-9-16s		280,909	39,441
	OldAK10-16s	AK administration	77,667	12,796
	OldAK11-16s		43,708	8,163
	OldAK12-16s		84,655	12,802
	OldAK13-16s		88,393	11,038
	OldAK14-16s		94,523	11,737
	OldAK15-16s		71,244	9,169
	OldAK6-16s		74,236	8,232
	OldAK8-16s		93,143	14,511
	OldV1-16s		84,869	10,111
	OldV1-2-16s		75,909	9,203
	OldV2-16s	Vehicle only	78,558	7,642
	OldV3-16s		1,392,223	140,351
	OldV3-2-16s		86,657	13,307
	OldV4-16s		99,173	13,187
	OldV4-2-16s		91,077	13,251
	OldV5-16s		60,100	7,125
	OldV6-16s		64,499	8,814
	OldV7-16s		84,257	10,306
	OldV8-16s		79,722	9,094

Library	Sample name	Sample	Raw data		Quality control (MG-RAST)		Gene prediction		
			Reads	Length (bp)	QC failed	QC passed	Ribosomal RNA gene	Reads with known functions	Reads with unknown functions
**Metagenome library**	A16	Aging (Week1)	3,560,386	343	509,738	3,050,648	28,649 (1%)	1,421,743 (49.00%)	1,451,378 (50.02%)
	A17		2,139,136	336	217,117	1,922,019	14,543 (1%)	822,163 (45.41%)	973,808 (53.79%)
	A6	Aging (Week4)	3,410,794	352	271,348	3,139,446	21,691 (1%)	2,358,242 (75.12%)	759,513 (24.19%)
	A7		4,121,549	340	1,719,921	2,401,628	19,479 (1%)	2,142,346 (89.20%)	239,803 (9.99%)
	A8		3,257,130	349	292,267	2,964,863	19,520 (1%)	2,504,308 (84.47%)	441,035 (14.88%)
	A9		3,494,225	344	359,280	3,134,945	22,700 (1%)	2,326,423 (74.21%)	785,822 (25.07%)
	A1	Aging (Week20)	2,530,032	332	208,669	2,321,363	15,230 (1%)	1,953,110 (84.14%)	353,023 (15.21%)
	A2		2,129,405	323	203,776	1,925,629	11,907 (1%)	1,657,137 (86.06%)	256,585 (13.32%)
	A3		3,571,464	337	446,637	3,124,827	22,161 (1%)	2,732,726 (87.45%)	369,940 (11.84%)
	A4		2,237,355	339	204,965	2,032,390	12,980 (1%)	1,734,577 (85.35%)	284,833 (14.01%)
	A5		6,104,820	339	997,235	5,107,585	39,944 (1%)	4,026,606 (78.84%)	1,041,035 (20.38%)
	A11	Aging (Week50)	2,851,025	344	305,916	2,545,109	16,459 (1%)	2,065,831 (81.17%)	462,819 (18.18%)
	A12		3,004,109	331	362,321	2,641,788	30,009 (1%)	1810062 (68.52%)	801717 (31.48%)
	A13		2,717,098	342	317,153	2,399,945	20,116 (1%)	2,068,043 (86.17%)	311,786 (12.99%)
	A14		3,255,758	340	366,123	2,889,635	22,518 (1%)	2,413,720 (83.53%)	453,397 (15.69%)
	A15		3,402,356	337	322,593	3,079,763	23,683 (1%)	2,632,194 (85.47%)	423,886 (13.76%)
	A24	Aging (Week100)	2,639,380	342	196,877	2,442,503	22,404 (1%)	1,943,896 (79.59%)	476,203 (19.50%)
	A25		3,199,177	347	322,089	2,877,088	25,079 (1%)	2,416,682 (84.00%)	435,327 (15.13%)
	A26		2,701,511	346	18,205	2,683,306	27,196 (1%)	1,826,371 (68.06%)	829,739 (30.92%)
	A27		3,204,270	345	378,430	2,825,840	26,685 (1%)	2,314,911 (81.92%)	484,244 (17.14%)
	A28		2,706,741	335	162,859	2,543,882	16,260 (1%)	2,043,088 (80.31%)	484,534 (19.05%)
	C3-1	Co-housing Co-Young	5,304,841	349	22,864	5,281,977	44,904 (1%)	3,789,442 (71.74%)	1,447,631 (27.41%)
	C3-2		6,434,506	350	1,145,390	5,289,116	35,780 (1%)	4,364,857 (82.53%)	888,479 (16.80%)
	C3-3		4,995,904	349	665,744	4,330,160	28,582 (1%)	3,434,243 (79.31%)	867,335 (20.03%)
	C3-4		4,110,449	345	585,607	3,524,842	29,042 (1%)	3,079,448 (87.36%)	416,352 (11.81%)
	C3-5		3,783,779	355	264,617	3,519,162	23,702 (1%)	2,869,856 (81.55%)	625,604 (17.78%)
	C3-6		3,919,226	353	26,310	3,892,916	37,793 (1%)	2,844,970 (73.08%)	1,010,153 (25.95%)
	C6-4		4,887,579	354	635,065	4,252,514	30,725 (1%)	3,527,593 (82.95%)	694,196 (16.32%)
	C6-5		3,636,200	344	444,662	3,191,538	21,751 (1%)	2,532,198 (79.34%)	637,589 (19.98%)
	C6-6		4,590,490	356	634,756	3,955,734	28,782 (1%)	3,383,726 (85.54%)	543,226 (13.73%)
	C3-10		3,879,336	343	563,100	3,316,236	22,988 (1%)	2,712,880 (81.81%)	580,368 (17.50%)

C3-20	Co-housing Co-Aged	4,019,805	349	450,110	3,569,695	24,463 (1%)	2,833,704 (79.38%)	711,528 (19.93%)
C3-30		4,293,989	342	355,692	3,938,297	26,848 (1%)	2,638,170 (66.99%)	1,273,279 (32.33%)
C3-40		5,207,190	356	715,748	4,491,442	32,847 (1%)	3,797,531 (84.55%)	661,064 (14.72%)
C3-50		3,371,812	339	495,800	2,876,012	23,001 (1%)	2,469,250 (85.86%)	383,761 (13.34%)
C3-60		4,117,093	355	672,978	3,444,115	27,561 (1%)	2,929,993 (85.07%)	486,561 (14.13%)
C6-40		4,769,955	355	564,531	4,205,424	28,499 (1%)	3,291,838 (78.28%)	885,087 (21.05%)
C6-50		4,753,125	354	791,629	3,961,496	28,559 (1%)	3,368,725 (85.04%)	564,212 (14.24%)
C6-60		3,582,024	350	366,398	3,215,626	21,477 (1%)	2,600,575 (80.87%)	593,574 (18.46%)
OY-Y1	Parabiosis Hetero-Young	2,180,580	340	472,234	1,708,346	16,239 (1%)	1,061,871 (62.2%)	630,236 (36.9%)
OY-Y2		2,917,992	332	271,454	2,646,538	15,703 (1%)	2,215,383 (83.71%)	415,452 (15.70%)
OY-Y3		3,058,229	353	170,662	2,887,567	10,957 (0%)	2,299,215 (79.62%)	577,395 (20.00%)
OY-Y4		3,729,589	355	424,488	3,305,101	18,008 (1%)	2,586,649 (78.26%)	700,444 (21.19%)
OY-O1	Parabiosis Hetero-Aged	3,097,182	352	249,950	2,847,232	13,719 (0%)	2,326,082 (81.70%)	507,431 (17.82%)
OY-O2		3,123,240	358	245,255	2,877,985	14,391 (1%)	2,312,513 (80.35%)	551,081 (19.15%)
OY-O3		3,679,412	337	454,741	3,224,671	21,110 (1%)	2,664,721 (82.64%)	538,840 (16.71%)
OY-O4		3,051,665	344	562,028	2,489,637	14,982 (1%)	2,169,167 (87.13%)	305,488 (12.27%)
YY1	Parabiosis Iso-Young	3,551,312	331	420,458	3,130,854	17,855 (1%)	2,592,275 (82.80%)	520,724 (16.63%)
YY2		4,122,268	339	590,168	3,532,100	22,458 (1%)	2,788,258 (78.94%)	721,384 (20.42%)
YY3		3,464,376	354	441,214	3,023,162	13,557 (0%)	2,540,286 (84.03%)	469,319 (15.52%)
YY4		2,867,284	346	426,913	2,440,371	15,759 (1%)	1,777,407 (72.83%)	647,205 (26.52%)
YY5		3,411,914	337	685,459	2,726,455	18,614 (1%)	2,435,826 (89.34%)	272,015 (9.98%)
YY6		2,583,837	341	396,743	2,187,094	12,199 (1%)	1,702,211 (77.83%)	472,684 (21.61%)
YY7		3,243,403	335	376,260	2,867,143	19,629 (1%)	2,400,790 (83.73%)	446,724 (15.58%)
YY8		2,869,815	346	200,834	2,668,981	17,195 (1%)	2,185,405 (81.88%)	466,381 (17.47%)
OO1	Parabiosis Iso-Aged	2,860,930	348	214,156	2,646,774	16,565 (1%)	2,084,371 (78.75%)	545,838 (20.62%)
OO2		3,427,569	347	303,219	3,124,350	17,744 (1%)	2,501,399 (80.06%)	605,207 (19.37%)
OO3		4,701,302	348	433,376	4,267,926	36,411 (1%)	2,742,438 (64.26%)	1,489,077 (34.89%)
OO4		3,142,834	343	796,861	2,345,973	14,313 (1%)	1,955,698 (83.36%)	375,962 (16.03%)
8iv_8_1	Serum-injection Young to Aged (iv8)	1,912,853	345	130,366	1,782,487	14,557 (1%)	1,477,597 (82.90%)	290,333 (16.29%)
8iv_8_2		2,472,280	354	174,082	2,298,198	18,245 (1%)	1,918,185 (83.46%)	361,768 (15.74%)
8iv_8_3		2,418,510	356	265,359	2,153,151	21,081 (1%)	1,870,437 (86.87%)	261,633 (12.15%)
16iv_8_4		2,340,729	359	372,729	1,968,000	14,624 (1%)	1,749,409 (88.89%)	203,967 (10.36%)
16iv_8_5		2,099,592	359	132,992	1,966,600	14,097 (1%)	1,576,877 (80.18%)	375,626 (19.10%)
8iv_8_12		2,672,682	360	321,469	2,351,213	22,648 (1%)	2,058,772 (87.56%)	269,793 (11.47%)
8iv_8_13		2,365,721	368	144,808	2,220,913	15,747 (1%)	1,837,940 (82.76%)	367,226 (16.53%)
16iv_8_14		2,436,675	366	231,289	2,205,386	15,633 (1%)	1,837,062 (83.30%)	352,691 (15.99%)
16iv_8_15		2,877,990	363	194,542	2,683,448	17,605 (1%)	2,232,463 (83.19%)	433,380 (16.15%)

16iv_8_16		2,271,201	374	153,746	2,117,455	14,798 (1%)	1,746,809 (82.50%)	355,848 (16.81%)
16iv_16_4	Serum-injection Young to Aged (iv16)	2,166,725	360	538,625	1,628,100	13,273 (1%)	1,333,534 (81.91%)	281,293 (17.28%)
16iv_16_5		2,421,420	358	162,291	2,259,129	15,935 (1%)	1,850,447 (81.91%)	392,747 (17.38%)
16iv_16_14		2,236,312	355	147,466	2,088,846	12,486 (1%)	1,725,304 (82.60%)	351,056 (16.81%)
16iv_16_15		1,921,825	353	170,866	1,750,959	13,151 (1%)	1,472,887 (84.12%)	264,921 (15.13%)
16iv_16_16		1,892,504	371	93,668	1,798,836	11,033 (1%)	1,513,903 (84.16%)	273,900 (15.23%)
Y-to-Y-8-1	Serum-injection Young to Young (iv8)	2,066,695	349	307,783	1,758,912	11,421 (1%)	1,492,265 (84.84%)	255,226 (14.51%)
Y-to-Y-8-2		2,179,868	353	191,875	1,987,993	14,337 (1%)	1,681,811 (84.60%)	291,845 (14.68%)
Y-to-Y-8-3		1,870,502	355	146,946	1,723,556	11,627 (1%)	1,384,635 (80.34%)	327,294 (18.99%)
Y-to-Y-8-4		1,895,983	352	184,783	1,711,200	10,563 (1%)	1,437,355 (84.00%)	263,282 (15.39%)
Y-to-Y-8-5		1,644,941	346	120,756	1,524,185	12,343 (1%)	1,240,560 (81.39%)	271,282 (17.80%)
Y-to-Y-8-6		1,946,742	346	218,461	1,728,281	13,029 (1%)	1,449,903 (83.89%)	265,349 (15.35%)
Y-to-Y-8-7		1,728,684	348	193,256	1,535,428	12,232 (1%)	1,321,475 (86.07%)	201,721 (13.14%)
Y-to-Y-8-8		1,532,361	347	95,417	1,436,944	10,725 (1%)	1,143,377 (79.57%)	282,842 (19.68%)
Y-to-Y-8-9		2,174,361	355	185,794	1,988,567	17,134 (1%)	1,663,136 (83.63%)	308,297 (15.50%)
Y-to-Y-8-11		2,156,026	357	143,179	2,012,847	13,179 (1%)	1,679,420 (83.44%)	320,248 (15.91%)
Y-to-Y-16-6	Serum-injection Young to Young (iv16)	1,722,630	356	110,209	1,612,421	14,114 (1%)	1,227,640 (76.14%)	370,667 (22.99%)
Y-to-Y-16-7		1,990,069	354	129,461	1,860,608	14,265 (1%)	1,547,912 (83.19%)	298,431 (16.04%)
Y-to-Y-16-8		1,811,045	349	101,826	1,709,219	12,348 (1%)	1,316,342 (77.01%)	380,529 (22.26%)
Y-to-Y-16-9		1,918,704	355	123,092	1,795,612	12,770 (1%)	1,405,767 (78.29%)	377,075 (21.00%)
22w-ak5	AK administration	2,183,447	353	138,544	2,044,903	15,219 (1%)	1,640,051 (80.20%)	389,633 (19.05%)
22w-ak6		1,514,569	350	69,671	1,444,898	9,783 (1%)	1,153,210 (79.81%)	281,905 (19.51%)
22w-ak8		1,669,728	347	74,075	1,595,653	9,434 (1%)	1,225,607 (76.81%)	360,612 (22.60%)
22w-ak9		1,702,860	351	89,830	1,613,030	9,527 (1%)	1,275,397 (79.07%)	328,106 (20.34%)
22w-ak10		1,323,462	354	66,282	1,257,180	8,074 (1%)	983,720 (78.25%)	265,386 (21.11%)
22w-ak11		1,602,441	352	68,439	1,534,002	11,785 (1%)	1,242,965 (81.03%)	279,252 (18.20%)
22w-ak12		1,597,264	351	98,369	1,498,895	10,684 (1%)	1,160,267 (77.41%)	327,944 (21.88%)
22w-ak13		1,538,080	354	81,352	1,456,728	9,733 (1%)	1,188,712 (81.60%)	258,283 (17.73%)
22w-ak14		1,478,840	348	96,728	1,382,112	9,211 (1%)	1,150,819 (83.27%)	222,082 (16.07%)
22w-ak15		1,853,018	360	105,009	1,748,009	13,007 (1%)	1,407,528 (80.52%)	327,474 (18.73%)
22w-v1-2	Vehicle only	1,810,233	343	93,578	1,716,655	12,916 (1%)	1,337,694 (77.92%)	366,045 (21.32%)
22w-v1		1,885,415	356	95,510	1,789,905	11,029 (1%)	1,415,062 (79.06%)	363,814 (20.33%)

22w-v2	2,029,327	353	135,408	1,893,919	15,546 (1%)	1,383,610 (73.06%)	494,763 (26.12%)
22w-v3-2	1,400,108	346	82,932	1,317,176	10,451 (1%)	1,047,458 (79.52%)	259,267 (19.68%)
22w-v3	1,951,734	333	137,928	1,813,806	9,242 (1%)	1,365,775 (75.30%)	438,789 (24.19%)
22w-v4-2	2,028,839	347	109,633	1,919,206	13,861 (1%)	1,546,140 (80.56%)	359,205 (18.72%)
22w-v4	1,369,663	351	80,941	1,288,722	8,110 (1%)	987,545 (76.63%)	293,067 (22.74%)
22w-v5	1,319,988	347	70,657	1,249,331	10,217 (1%)	1,014,900 (81.24%)	224,214 (17.95%)
22w-v6	1,621,916	348	107,813	1,514,103	10,766 (1%)	1,206,625 (79.69%)	296,712 (19.60%)
22w-v7	1,733,476	346	82,226	1,651,250	11,178 (1%)	1,298,632 (78.65%)	341,440 (20.68%)
22w-v8	1,454,574	347	81,121	1,373,453	9,221 (1%)	1,075,752 (78.32%)	288,480 (21.00%)
