# Supplementary material for: Ageing and rejuvenation models reveal changes in key microbial communities associated with healthy ageing
Source: Microbiome. 2021 Dec 15;9:240. doi: 10.1186/s40168-021-01189-5 (PMC8672520; doi:10.1186/s40168-021-01189-5)
Supplement: Supplementary file 6 — Additional file 5: Table S4. Effect of parabiotic pairing on blood parameters. [file 40168_2021_1189_MOESM6_ESM.pdf]

Table S4. Effect of parabiotic pairing on blood parameters.

	AST (IU/L)	ALT (IU/L)	ALP (IU/L)	BUN (mg/dl)	Crea (mg/dl)	Chol (mg/dl)	TG (mg/dl)
Iso-Y	139±11.8	45±8.6	56±5.3^{b}	36±5.2	0.2±0.03^{a}	122±4.3^{ab}	78±16.1
Hetero-Y	123±15.2	50±8.5	65±5.3^{ab}	32±2.1	0.2±0.01^{a}	113±3.3^{b}	81±16.8
Hetero-A	116±18.9	63±11.6	73±2.0^{a}	39±3.5	0.1±0.02^{b}	129±6.0^{a}	52±6.0
Iso-A	157±25.6	66±10.7	72±5.5^{a}	32±5.0	0.2±0.03^{ab}	100±2.7c	56±3.8

^{abc} Levels not connected by same letter are significantly different.
