# Supplementary material for: Ageing and rejuvenation models reveal changes in key microbial communities associated with healthy ageing
Source: Microbiome. 2021 Dec 15;9:240. doi: 10.1186/s40168-021-01189-5 (PMC8672520; doi:10.1186/s40168-021-01189-5)
Supplement: Supplementary file 9 — Additional file 8: Table S7. Frailty score used to develop a clinical frailty index in mice. [file 40168_2021_1189_MOESM9_ESM.pdf]

Table S7. Frailty score used to develop a clinical frailty index in mice.

	Parameter
Integument	Alopecia<br>Loss of fur colour<br>Dermatitis<br>Loss of whiskers<br>Coat condition
Physical/Musculoskeletal	Tumours<br>Distended abdomen<br>Kyphosis<br>Tail stiffening<br>Gait<br>Tremor<br>Forelimb grip strength
Vestibulocochlear/Auditory	Head tilt<br>Hearing loss<br>Cataracts<br>Eye discharges/swollen/squint<br>Microphthalmia<br>Corneal opacity<br>Vision loss<br>Menace reflex<br>Nasal discharges
Digestive/Urogenital	Malocclusions<br>Rectal prolapse<br>Penile/Uterine prolapse<br>Diarrhoea
Respiratory	Breathing rate/depth
Discomfort	Piloerection
