# Supplementary material for: Ageing and rejuvenation models reveal changes in key microbial communities associated with healthy ageing
Source: Microbiome. 2021 Dec 15;9:240. doi: 10.1186/s40168-021-01189-5 (PMC8672520; doi:10.1186/s40168-021-01189-5)
Supplement: Supplementary file 10 — Additional file 9: Table S8. Sequences of PCR primers used in this study. [file 40168_2021_1189_MOESM10_ESM.pdf]

Table S8. Sequences of PCR primers used in this study

Gene	Gene Bank Accession Number	Primer Sequence
Zo-1	NM_001329965.1	Forward 5’-TTCCTGAACCACAGCGAGAC-3’<br>Reverse 5’-GGTTACAAGCACCCTCTGCT-3’
Ocln	NM_008756.2	Forward 5’-TTGGGACAGAGGCTATGGGA-3’<br>Reverse 5’-AAGCGATGAAGCAGAAGGCT-3’
Cldn1	NM_016674.4	Forward 5’-GGCTTCTCTGGGATGGATCG-3’<br>Reverse 5’-TTTGCGAAACGCAGGACATC-3’
Cldn3	NM_009902.4	Forward 5’-TACAAGACGAGACGGCCAAG-3’<br>Reverse 5’-GGGCACCAACGGGTTATAGA-3’
Cldn4	NM_009903.2	Forward 5’-TTCTCTCAGTGGTAGGGGGC-3’<br>Reverse 5’-ACGGGCACCATAATCAGCAT-3’
Muc2	NM_023566.3	Forward 5’-CCTGAAGACTGTCGTGCTGT-3’<br>Reverse 5’-GGGTAGGGTCACCTCCATCT-3’
Muc3	NM_010843.2	Forward 5’-GCTGGCTTTCATCCTCCACT-3’<br>Reverse 5’-GCTGTCGTCTTGGGTGCTAT-3’
Lgr5	NM_010195.2	Forward 5’-CAGGTCAATACCGGAGCGAG-3’<br>Reverse 5’-GCGAGGCACCATTCAAAGTC-3’
Bmi1	NM_007552.4	Forward 5’-TGCTGGAGAGCTGGAAAGTG-3’<br>Reverse 5’-GTGAGGGAACTGTGGGTGAG-3’
Hopx	NM_175606.3	Forward 5’-ACCAGGTGGAGATCCTGGAGTA-3’<br>Reverse 5’-CCAGGCGCTGCTTAAACCAT-3’
Ascl2	NM_008554.3	Forward 5’-CACCAGAACTCGTAGCAGGG-3’<br>Reverse 5’-AGCAATTCCGGAGACGCTTG-3’
Bcatenin	NM_007614.3	Forward 5’-CCCAGTCCTTCACGCAAGAG-3’<br>Reverse 5’-CATCTAGCGTCTCAGGGAACA-3’
Myc	NM_001177352.1	Forward 5’-GCTGTTTGAAGGCTGGATTTC-3’<br>Reverse 5’-GATGAAATAGGGCTGTACGGAG-3’
Ephb2	NM_001290753.2	Forward 5’-ACTATGGCGGCTGTATGTCC-3’<br>Reverse 5’-GCACATCCACTTCTTCAGCA-3’
CD44	NM_009851.2	Forward 5’-GTGGGCAGAAGAAAAAGCTG-3’<br>Reverse 5’-TGATGGTTCCTTGTTCACCA-3’
Axin2	NM_015732.4	Forward 5’-CTCCCCACCTTGAATGAAGA-3’<br>Reverse 5’-ACT GGG TCG CTT CTC TTG AA-3’
Olfm4	NM_001351947.1	Forward 5’-GCCAGATCTTGGCTCTGAAG-3’<br>Reverse 5’-GCCAGTTGAGCTGAATCACA-3’
CyclinD1	NM_007631.2	Forward 5’-CCAGCTCCTGTGCTGCGAAG-3’<br>Reverse 5’-CATGGATGGCACAATCTCCT-3’
Wnt3	NM_009521.2	Forward 5’-CAAGCACAACAATGAAGCAGGC-3’<br>Reverse 5’-TCGGGACTCACGGTGTTTCTC-3’
